# Antiviral Activities of Compounds Isolated from *Pinus densiflora* (Pine Tree) against the Influenza A Virus

**DOI:** 10.3390/biom10050711

**Published:** 2020-05-04

**Authors:** Thi Kim Quy Ha, Ba Wool Lee, Ngoc Hieu Nguyen, Hyo Moon Cho, Thamizhiniyan Venkatesan, Thi Phuong Doan, Eunhee Kim, Won Keun Oh

**Affiliations:** 1 Korea Bioactive Natural Material Bank, Research Institute of Pharmaceutical Sciences, College of Pharmacy, Seoul National University, Seoul 08826, Korea; htkquy@ctu.edu.vn (T.K.Q.H.); paul36@snu.ac.kr (B.W.L.); hieusnu@gmail.com (N.H.N.); chgyand@naver.com (H.M.C.); thamtris@snu.ac.kr (T.V.); phuongdoan@snu.ac.kr (T.P.D.); 2 College of Natural Sciences, Cantho University, Campus II, Cantho City 94000, Vietnam; 3 Choong Ang Vaccine Laboratory, 1476-37, Yuseong-daero, Yuseong-gu, Daejeon 34055, Korea; ehkim@cavac.co.kr

**Keywords:** *Pinus densiflora*, anti-influenza, neuraminidase, H1N1, cytopathic effect, anti-inflammation

## Abstract

*Pinus densiflora* was screened in an ongoing project to discover anti-influenza candidates from natural products. An extensive phytochemical investigation provided 26 compounds, including two new megastigmane glycosides (**1** and **2**), 21 diterpenoids (**3–23**), and three flavonoids (**24–26**). The chemical structures were elucidated by a series of chemical reactions, including modified Mosher’s analysis and various spectroscopic measurements such as LC/MS and 1D- and 2D-NMR. The anti-influenza A activities of all isolates were screened by cytopathic effect (CPE) inhibition assays and neuraminidase (NA) inhibition assays. Ten candidates were selected, and detailed mechanistic studies were performed by various assays, such as Western blot, immunofluorescence, real-time PCR and flow cytometry. Compound **5** exerted its antiviral activity not by direct neutralizing virion surface proteins, such as HA, but by inhibiting the expression of viral mRNA. In contrast, compound **24** showed NA inhibitory activity in a noncompetitive manner with little effect on viral mRNA expression. Interestingly, both compounds **5** and **24** were shown to inhibit nitric oxide (NO) production and inducible nitric oxide synthase (iNOS) expression in a dose-dependent manner. Taken together, these results provide not only the chemical profiling of *P. densiflora* but also anti-influenza A candidates.

## 1. Introduction

According to data from the Centers for Disease Control and Prevention (CDC), it is estimated that *5*–10% of the human population is infected by seasonal human influenza virus each year, and approximately 290,000 to 650,000 people globally die from influenza-related respiratory diseases [1,2]. The influenza virus is divided into four types (A-D) based on the core protein, and the influenza A virus (IAV) is considered the main target for drug development, especially since it is the most devastating in pandemic or epidemic outbreaks [3]. Many efforts have been made to develop anti-influenza A agents that inhibit each key step of IAV from various resources. Major events that occur in viral replication of IAV are as follows: (i) attachment of a virion by interaction between the sialic acid receptor of the host cell and hemagglutinin (HA) on the virion surface; (ii) fusion and endocytosis of the virion into the cytoplasm of the host cell; (iii) release of viral ribonucleoprotein (RNP) into the cytoplasm and nuclear import; (iv) transcription and replication of viral RNA; (v) translation and protein synthesis; (vi) assembly of virion composition; and (vii) budding and release of a newly assembled virion by neuraminidase [4].

Pharmaceutical companies have endeavored to develop inhibitors that block any of these aforementioned steps. Among these targets, blocking viral entry and fusion has been emerging as a favorable inhibition target because hemagglutinin, especially, HA2 subunit (stem region of HA) responsible for viral fusion is highly conserved and able to trigger an antibody-mediated immune response during infection [5,6], in which the antibodies induced by HA2 show high cross-reactivity, even toward various different subtypes of influenza virus [7]. As a result, several potent HA inhibitors including cholesterol conjugated short peptide named S-KKWK, chlorogenin 3-*O*-*β*-chacotrioside derivatives, and fucoidan derivatives have been investigated to date [8,9,10]. For clinically approved drugs, umifenovir interrupts viral fusion by targeting viral HA and is being used as an over-the-counter (OTC) drug in Russia and China [11]. Interestingly, although umifenovir has been used clinically for more than 30 years in Russia, no reports on emergence of resistant viruses from humans have been published so far [12]. DAS181, which prevents binding of the influenza virus by cleaving viral receptors on host epithelical cells, is in phase III clinical trials [13]. Amantadine and rimantadine have been used to inhibit the release of viral RNP as M2 ion channel inhibitors. However, most influenza strains have developed resistance to both drugs [14]. Neuraminidase inhibitors, including zanamivir and oseltamivir, which are most commonly prescribed to treat flu, have been used globally. However, their use has been facing limitations due to the rapid emergence of resistant influenza strains and the risk of side effects [15]. Therefore, the discovery of small molecules that inhibit any step of the virus life cycle and can be used in combination to prevent the rapid occurrence of resistant viruses is still urgent.

*Pinus densiflora* Siebold and Zucc., commonly known as Korean red pine, is a coniferous evergreen tree with an irregular or umbrella-shaped crown that can grow up to 100 feet tall. *P. densiflora* belongs to the Pinaceae family and is widely distributed in East Asia, including the Russian Far East, Northern China, Central and Southern Japan, and Korea [16]. Various parts of *P. densiflora,* such as the needles, resins and pollen, have been listed in two famous ancient pharmacopoeias, Dong-Eu-Bo-Gam (Heo, Joon, AD 1713) [17] of Korea and the Compendium of Materia Medica of China (Li, Shizhen, 1578) [18]. Its resin and pollen as medicinal raw materials can still be found in the Korean Herbal Pharmacopoeia [19]. *P. densiflora* has been established as having health-promoting properties, including the treatment of strokes and memory improvement as a nourishing tonic [20,21]. In addition, various activities such as the improvement of fatigue, depression, anxiety, cancer, and several chronic diseases have been reported [22,23]. With respect to antiviral properties, potent inhibitory activity of some fractions from *P. densiflora* against HSV (herpes simplex virus) has been suggested experimentally [24]. However, to the best of our knowledge, there have been limited studies evaluating the antiviral properties of *P. densiflora*, especially against influenza A at the compound level. Regarding its chemical components, essential oils (0.3–1.3%, antioxidant and antiaging) including *α*-pinene, *β*-pinene, camphene, limonene, borneol (6.8%), bornyl acetate (3.8%), along with cinnamic acid, benzoic acid, flavonoids, abietane diterpenoid, and stilbenoid (antibacterial and antifungal) have been reported from its leaves [20,25,26,27]. It has been reported that the pine cones that contain labdane-type diterpenoids that have inhibitory activity against acne [28]. Extensive isolation of the compounds from *P. densiflora* allowed the purification of 26 compounds with various skeletons, including two previously undescribed megastigmane glycosides (**1** and **2**), which contributes to the chemical profiling of constituents in *P. densiflora*. Moreover, all isolated compounds were tested by a cytopathic effect (CPE) assay with H1N1 virus infection, which is an indicator of the potential candidates as being anti-influenza agents. For selected active candidates, various assays including, Western blot, immunofluorescence assay, flow cytometry, and NO production assay, were performed to investigate the anti-influenza mode of action.

## 2. Materials and Methods

### 2.1. General Experimental Procedures

Optical rotation values were obtained using a JASCO P-2000 polarimeter (JASCO International Co. Ltd., Tokyo, Japan). IR spectra were acquired using a Nicolet 6700 FT-IR spectrometer (Thermo Electron Corp., Waltham, MA, USA). NMR spectra were recorded on an Advance 500 MHz spectrometer (Bruker, Billerica, MA, USA). HRESIMS data were obtained using an Agilent 6530 Q-TOF (Agilent Technologies, Inc., Santa Clara, CA, USA). Column chromatography (CC) was performed with silica gel (63–200 μm particle size, Zeochem, Lake Zurich, Switzerland) and RP-C_18_ (75 μm particle size, Nacalai Tesque, Kyoto, Japan). For TLC analysis, RP-18 F_254_S and silica gel 60 F_254_ plates, from Merck (Darmstadt, Germany) were used. A Gilson HPLC system equipped with an Optima Pak C18 column (10 × 250 mm, 10 μm particle size; RS Tech, Seoul, Korea) was used for the purification of compounds, with a flow rate of 2 mL/min and UV detection at 205 and 254 nm. Industrial-grade solvents (Daejung Chemical, Siheung, Korea) were used for preprocessing, including extraction and fractionation, and analytical-grade solvents (Daejung Chemical) were used for purification of the compounds.

### 2.2. Plant Material

The cortex and leaves of *P. densiflora* were collected from the medicinal plant garden of the College of Pharmacy, Seoul National University, Goyang-si, Gyeonggi-do, Korea (37°71′27′N, 126°81′88′E). The samples were botanically authenticated by Prof. Won Keun Oh at the College of Pharmacy, Seoul National University. A voucher specimen (SNUPMHG-001837) was deposited at the College of Pharmacy, Seoul National University, Seoul, Republic of Korea.

### 2.3. Extraction and Isolation 

The cortex (1.5 kg) and leaves (2.5 kg) of *P. densiflora* were extracted separately with MeOH with the assistance of ultrasonic waves. The combined extracts were then evaporated under reduced pressure to obtain the crude residue. The crude extracts were dispensed in distilled water and successively partitioned with EtOAc and *n*-BuOH. The EtOAc and *n*-BuOH-soluble fractions were then fractionated and purified by repeated normal-phase and reverse-phase chromatography, as well as by preparative HPLC, resulting in the isolation of 26 compounds, including two new megastigmane-type compounds **(1** and **2),** 21 diterpenoids, and three flavonoids. The isolation scheme and NMR spectra are reported in the Supplementary Material (Figures S1 and S2). All compounds in this study were confirmed to have high purity (≥ 95%). 

(2*S*,9*S*)-2,9-Dihydroxymegastigman-5-ene-2-*O*-*β*-d-glucopyranoside (1): Colorless gum; ${\left[\alpha \right]}_{D}^{20}$-14.1 (*c* = 0.5, MeOH); IR *ν*_max_ 3360, 2969 cm^−1^; HRESIMS *m/z* 419.2282 [M+HCOO]^−^ (calcd for C_20_H_35_O_9_, 419.2287); ^1^H and ^13^C NMR see Table 1. 

(2*R*,9*S*)-2,9-Dihydroxymegastigman-5-ene-2-*O*-*β*-d-glucopyranoside (2): Colorless gum; ${\left[\alpha \right]}_{D}^{20}$-4.7 (*c* = 0.5, MeOH); IR *ν*_max_ 3358, 2969 cm^−1^; HRESIMS *m/z* 419.2292 [M+HCOO]^−^ (calcd for C_20_H_35_O_9_, 419.2287); ^1^H and ^13^C NMR see Table 1.

### 2.4. Enzymatic Hydrolysis of ***2***

A solution of **2** (4.4 mg) in 20 mM acetate buffer (approximately pH 5.0, 0.5 mL) was treated with *β*-glucosidase from almonds (10.1 mg), and the solution was incubated at 37 °C for 24 h [29]. The mixture was further incubated after the addition of *β*-glucosidase (5.3 mg) for 28 h. The reaction mixture was dried under a N_2_ stream after cooling and subjected to silica gel column chromatography (16.5 g, Φ = 15 mm, *L* = 25 cm). The residue was eluted from *n*-hexane and acetone (3/1) to methanol. The aglycone **2a** (1.2 mg) was obtained in fractions 18–20. Aglycone (**2a**): Colorless syrup, ${\left[\alpha \right]}_{D}^{20}$+15.3 (*c* = 0.2, CHCl_3_); ^1^H-NMR (CH_3_OD, 400 MHz) *δ*: 3.41 (dd, *J* = 10.0, 3.6 Hz, H-2), 1.71 (m, H-3), 2.03 (overlapped, H-4), 2.20 (m, H-7a), 1.95 (m, H-7b), 1.50 (m, H-8), 3.71 (m, H-9), 1.17 (d, *J* = 6.4 Hz, H-10), 0.96 (s, H-11), 1.07 (s, H-12), 1.61 (s, H-13); ^13^C-NMR (CH_3_OD, 100 MHz) *δ*: 41.3 (C-1), 76.9 (C-2), 27.9 (C-3), 31.5 (C-4), 127.1 (C-5), 137.3 (C-6), 26.1 (C-7), 40.7 (C-8), 69.2 (C-9), 23.3 (C-10), 21.8 (C-11), 26.4 (C-12), 19.8 (C-13); HRESIMS (positive-ion mode): *m/z*: 213.1853 [M+H]^+^ (calcd C_13_H_25_O_2_: 213.1849) (Figures S13–S16).

### 2.5. Preparation of 2,9-Di-(S)-MTPA Ester (***2b***) and 2,9-Di-(R)-MTPA Ester (***2c***) from ***2a***

The absolute configuration of C-9 in **2** was determined by modified Mosher’s analysis [30]. After transferring **2a** (0.4 mg, 1.9 μmol) in dry pyridine (4.8 μL, 60 μmol), 0.5 mL of anhydrous CH_2_Cl_2_ was added to the mixture for dissolution. Then, *R*-(–)-MTPA-Cl (15 μL, 78.3 μmol) was added to the mixture, and the reaction was initiated at ambient temperature for 6 h. The progress of the reaction was monitored by TLC using *n*-hexane and acetone (3:1). After the reaction was complete, the resulting solution was dried under a N_2_ stream and dissolved in distilled water, followed by repeated partitioning with CH_3_Cl. The combined CH_3_Cl extracts containing 2,9-di-(*S*)-MTPA ester (**2b)** were dried, and ^1^H-NMR and ^1^H-^1^H COSY were recorded in CDCl_3_. 2,9-Di-(*R*)-MTPA ester (**2c**) was also prepared in a similar manner as **2b**. Compound **2b**: ^1^H-NMR (600 MHz, CDCl_3_) *δ*: 4.95 (dd, *J* = 9.6, 3.0 Hz, H-2), 1.83-1.91 (m, H-3), 1.99-2.06 (overlapped, H-4), 2.05 (overlapped, H-7a), 1.93 (overlapped, H-7b), 1.66 (m, H-8a), 1.60 (m, H-8b), 5.10 (m, H-9), 1.28 (d, *J* = 6.0, H-10), 0.89 (s, H-11), 0.91 (s, H-12), 1.54 (s, H-13); HR-ESI-MS (positive-ion mode): *m/z*: 662.2899 [M + NH_4_]^+^ (calcd C_33_H_42_F_6_NO_6_: 662.2911) (Figure S17). Compound **2c**: ^1^H-NMR (600 MHz, CDCl_3_) *δ*: 4.89 (dd, *J* = 9.0, 3.0 Hz, H-2), 1.77-1.85 (m, H-3), 1.92-1.98 (overlapped, H-4), 1.92 (overlapped, H-7a), 1.88 (overlapped, H-7b), 1.61 (m, H-8a), 1.56 (m, H-8b), 5.13 (m, H-9), 1.35 (d, *J* = 6.6, H-10), 0.87 (s, H-11), 0.92 (s, H-12), 1.46 (s, H-13); HR-ESI-MS (positive-ion mode): *m/z*: 662.2911 [M + NH_4_]^+^ (calcd C_33_H_42_F_6_NO_6_: 662.2911) (Figure S18).

### 2.6. Sugar Analysis of Compounds ***1*** and ***2***

The absolute configuration of the sugars was determined by means of HPLC analysis after derivatization, which was proposed by Tanaka [31]. A solution of **1** (0.6 mg) in 2 M hydrochloric acid was incubated at 100 °C for 4.5 h. The hydrolysates were neutralized with Na_2_CO_3_ and concentrated under a N_2_ stream. l-Cysteine methyl ester (0.6 mg) in pyridine was added to the dried residue and subjected to incubation at 60 °C for 1 h. Then, 0.6 mg of phenyl isothiocyanate was added to the resulting mixture and incubated under the same conditions for 1 h. After cooling and filtration, 10 μL of the solution was directly analyzed by reverse-phase HPLC [HPLC: Thermo Fisher Ultimate 3000 system (Germering, Germany); YMC Triart C18 column (4.6 × 250 mm, 5 μm particle size; YMC Co., Kyoto, Japan); conditions were as follows: MeCN: H_2_O = 25: 75 (*v:v*); a diode array detector at 250 nm; and a flow rate of 0.8 mL/min]. The UV pattern and retention time of the derivatized sugar in compound **1** were compared to those of derivatized authentic d,l-glucose. The absolute configuration of the sugar in compound **2** was determined in a similar manner. The retention time of derivatives of d-glucose is 19.753 min, whereas that of l-glucose is 17.663 min. As derivatives of compounds **1** and **2** were eluted at 19.767 min and 19.800 min, respectively, the sugar moieties of both **1** and **2** were proven to be in the d-configuration.

### 2.7. Cell Culture and Virus Stock

Madin–Darby Canine Kidney (MDCK) cells and RAW264.7 cells were provided by American Type Culture Collection (ATCC, Manassas, VA 20108, USA). The cells were maintained in Dulbecco’s modified Eagle’s medium (DMEM) (HyClone, Logan, UT) supplemented with 10% fetal bovine serum (FBS) (HyClone, Logan, UT), 100 U/mL penicillin and 100 μg/mL streptomycin (GIBCO-BRL, Grand Island, NY, USA). Influenza viruses (H1N1 A/PR/8/34 virus, H9N2 A/chicken/Korea/01210/2001 virus) were obtained from Choong Ang Vaccine Laboratory, Korea and stored at −80 °C.

### 2.8. Cytopathic Effect (CPE) Inhibition Assay

Cytopathic effect and cytotoxicity assays were performed as previously described [32]. Briefly, MDCK cells were grown in 96-well plates for 24 h. The cells were inoculated with influenza viruses (H1N1 A/PR/8/34 virus or H9N2 A/chicken/Korea/01210/2001 virus) at 0.01 MOI using DMEM containing 0.15 µg/mL trypsin and 5 µg/mL bovine serum albumin (BSA) (Sigma, St Louis, MO, USA). After 2 h of incubation, the cells were washed with phosphate-buffered saline (PBS) (TaKaRa, Japan) and the medium was replaced with new medium containing the test compounds. The cells were continually incubated for 3 days at 37 °C under a 5% CO_2_ atmosphere. Then, the medium was replaced with fresh DMEM, and 20 µL of 2 mg/mL 3-(4,5-dimethyl-2-thiazolyl)-2,5-diphenyl-2*H*-tetrazolium bromide (MTT) solution (Sigma, St Louis, MO, USA) was added to each well. After 4 h of incubation, the supernatant was removed and formazan crystals were dissolved in 100 µL of DMSO. The absorbance was measured at a wavelength of 550 nm. Similarly, the cytotoxicity assay was carried out using 96-well plates. After 24 h of incubation, MDCK cells were washed with PBS and treated with the test compounds. The culture was treated with 20 µL of 2 mg/mL MTT solution in each well after 2 days of incubation. The final steps followed those described above.

### 2.9. Quantitative Real-Time PCR

MDCK cells were maintained in 6-well plates and infected with the H1N1 A/PR/8/34 virus for 2 h. The cells were washed twice with PBS and the medium was replaced with new media containing the test compounds. The TRIzol method was used to isolate total RNA from the cells after 24 h of incubation [33]. Total RNA was reverse transcribed using random primers (Invitrogen, USA) according to the manufacturer’s instructions. Real-time PCR was performed using selective primers for H1N1 (Table S5) (Enotech, Korea) using the Maxima SYBR Green qPCR master mix 2X kit (Thermo Sci., Rockford, IL, USA). The StepOnePlus^TM^ real-time PCR system was used with cycling conditions as follows: 95 °C for 10 min, followed by 40 cycles of 95 °C for 15 sec and 60 °C for 1 min. The data were calculated using StepOne software v2.3 (Applied Biosystems).

### 2.10. Western Blotting Analysis

The cultures were prepared similarly to the cultures prepared for the quantitative real-time PCR assay. However, after 24 h of incubation, the cells were washed with cold PBS and stored at −80 °C until use. The cells were then lysed with lysis buffer [50 mM Tris-HCl (pH 7.6), 120 mM NaCl, 1 mM EDTA, 0.5% NP-40, and 50 mM NaF]. The protein concentrations of the lysates were determined using a protein assay kit (Bio-Rad Laboratories Inc., USA) and electrophoresed on 12% SDS-polyacrylamide gels [34]. The gels were electrotransferred to polyvinylidene fluoride membranes (PVDF 0.45 µm, Immobilon-P, USA). The membranes were blocked with a 5% skim milk solution (Becton Dickinson, USA) and then incubated with primary antibodies: neuraminidase (NA; Gene Tex, San Antonio, TX, USA), hemagglutinin (HA; Sigma, St Louis, MO, USA) or mouse monoclonal actin (Abcam, Cambridge, UK). After overnight incubation, the membranes were further incubated with secondary antibodies (Goat anti-Rabbit IgG (H + L) HRP or Goat anti-Mouse IgG (H + L) HRP; Thermo Sci., Rockford, IL, USA) for 2 h and detected by a chemiluminescence Western blotting detection kit (Thermo Sci., Rockford, IL, USA) using Image Reader LAS-4000 software.

### 2.11. Immunofluorescence Assay

MDCK cells were grown on sterilized glass coverslips for 24 h. Then, the cells were infected with the H1N1 A/PR/8/34 virus for 2 h, washed twice with PBS and the medium was replaced with new media containing the test compounds. The cultures were continually incubated at 37 °C under a 5% CO_2_ atmosphere for 24 h. After washing with PBS (pH 7.4), the cells were fixed with a 4% paraformaldehyde solution for 30 min at room temperature and blocked with a 1% BSA solution for 1 h. The cells were incubated overnight with a rabbit monoclonal antibody against NA (GeneTex, San Antonio, TX, USA) diluted 1:50 in PBS (pH 7.4). After being washed with PBS, the cells were incubated with a secondary FITC-conjugated Goat anti-Rabbit IgG antibody (Abcam, Cambridge, UK). After washing with PBS (pH 7.4) [34], the cells were stained with 500 nM DAPI solution for 10 min at room temperature and washed again with PBS (pH 8.0). Slides were mounted and imaged by fluorescence microscopy (Olympus ix70 Fluorescence Microscope, USA).

### 2.12. Flow Cytometric Analysis of the Cell Cycle

MDCK cells were maintained in 36-mm culture dishes and infected with the H1N1 A/PR/8/34 virus for 2 h. After washing with PBS and replacing the media with new media containing the test compounds, the cultures were incubated for 3 days at 37 °C under a 5% CO_2_ atmosphere. The cell suspensions were collected from adherent cells and detached cells. After that, the cells were washed with cold PBS and fixed with precooled 70% ethanol solution. After overnight incubation at 4 °C, the cells were stained with 20 µg/mL PI (propidium iodide) in PBS solution for 30 min at room temperature [35]. The cellular DNA contents were analyzed using a flow cytometer (BD, FACSCalibur, San Jose, CA, USA). Data were collected for at least 10,000 cells per sample. In each phase of the viral-infected cell cycle, the distribution of cells is displayed using histograms.

### 2.13. Neuraminidase Inhibition and Kinetic Assays 

NA inhibition and kinetic assays were performed as previously described [32,36]. In general, NA activity was measured using 2′-(4-methylumbelliferyl)-*α*-D-N-acetylneuraminic acid (4-MU-NANA) (Sigma-Aldrich Co., USA) as the fluorescent substrate in 96-well plates. Briefly, 10 μL of test compounds diluted in enzyme buffer (32.5 mM 2-(*N*-morpholino) ethanesulfonic acid, 4 mM CaCl_2_, pH 6.5) and 10 μL of virus suspension containing NAs from four subtypes (H1N1 A/PR/8/34 virus, H9N2 A/chicken/Korea/01210/2001 virus, wild-type (wt) pandemic 2009 H1N1 virus (A/California/07/2009), oseltamivir-resistant virus (H274Y mutation) were coincubated for 30 min at 37 °C. Then, 30 μL of 42 μM 4-MU-NANA substrate in enzyme buffer was added to each well. The enzymatic reactions were maintained at 37 °C for 2 h and quenched with 150 μL of stop solution (25% EtOH, 0.1 M glycine, pH 10.7). The fluorescence was measured with an excitation wavelength of 360 nm and an emission wavelength of 440 nm using a SpectraMax GEMINI XPS microplate reader (Molecular Devices, Sunnyvale, CA, USA). The enzymatic kinetic assay was performed in a similar manner as that of the NA inhibition assay. The product 4-methylumbelliferone was measured immediately without adding stop solution. The inhibition type was analyzed using SigmaPlot 11.0 (SPCC Inc., Chicago, IL, USA). 

### 2.14. Cell Protection Assay for H1N1 Infection via Coincubation

In order to evaluate the effect of coincubation of compounds **5** and **24** with the host cells, MDCK cells were preincubated with the two test compounds (20 μM) for 4 h at 37 °C, followed by H1N1 A/PR/8/34 virus infection [37]. After 1 h incubation at 37 °C under a 5% CO_2_ atmosphere, the cells were washed twice with PBS. Then, the medium was replaced with fresh medium and the cells were incubated for 3 days. The antiviral activity was evaluated by a cytotoxicity assay.

### 2.15. Virus Particles Assay for H1N1 Infection via Coincubation

In order to evaluate the effect of coincubation of compounds **5** and **24** with virus regarding its infectivity, H1N1 A/PR/8/34 virus diluted with DMEM containing 0.15 μg/mL trypsin and 5 μg/mL BSA was incubated with or without the test compounds (20 μM) for 1 h at 4 °C [37]. Then, MDCK cells were incubated with the viral medium for 1 h at 37 °C under a 5% CO_2_ atmosphere. The monolayers of cells were washed twice with PBS and incubated for 3 days in new medium. The antiviral activity was evaluated by a cytotoxicity assay.

### 2.16. Nitric Oxide (NO) Production Assay

DMEM without phenol red (WelGene, Korea) was used for the NO production assay. Briefly, RAW 264.7 cells were seeded into 12-well plates and incubated for 1 day at 37 °C under a 5% CO_2_ atmosphere. The cells were treated with the test compounds for 10 h before infection with the H1N1 A/PR/8/34 virus at high MOI (1.0) and low MOI (0.01) for 1 h [38]. The medium was replaced with new medium and the cells were further incubated for 12 h. Nitric oxide production was evaluated by using the Griess reagent method, and the absorbance was measured at 540 nm [39]. For the NO assay stimulated by lipopolysaccharide (LPS), RAW 264.7 cells were pretreated with the test compounds for 10 h, and 100 ng/mL LPS was added to the cells. After 12 h of incubation, NO production was measured as described above. Inducible nitric oxide synthase (iNOS) expression was measured by Western blot. Briefly, RAW 264.7 cells were maintained in 6-well plates for 24 h. The cells were preincubated with test compounds for 10 h, and then 100 ng/mL LPS was added to the culture. The cells were collected after 12 h of incubation. The Western blot method was performed as described above with a monoclonal iNOS antibody (Thermo Sci., Rockford, IL, USA).

### 2.17. Simulation of Binding Affinity via Molecular Docking

In order to evaluate the binding affinity between the ligands and influenza viral polymerase or NA in silico, the docking study using Discovery Studio 4.0/CDOCKER (Accelrys, San Diego, CA) was performed [40]. The crystal structure of the influenza A virus nucleoprotein (NP; PDB ID: 3RO5), the PA-PB1 complex from influenza virus RNA polymerase (PDB ID: 2ZNL), the 2009 pandemic H1N1 NA complex (PDB ID: 3TI4), and the N1 NA H274Y (PDB ID: 3CL0) were supplied from the Protein Data Bank (http://www.pdb.org) [41,42,43,44]. Ligand interaction affinity was calculated by CDOCKER interaction energy and interacting bonds such as van der Waals, salt bridge, attractive charge, conventional hydrogen bond, carbon hydrogen bond, pi-cation, pi-anion, pi-alkyl, and pi-pi T-shaped bonds.

### 2.18. Statistical Analysis

The results were obtained from three independent experiments. Statistical calculations were conducted with Sigma Plot Statistical 11.0, SPSS Statistics 23, StepOne software v2.3, and a LAS 4000 luminescent image analyzer. The results were calculated as the means ± SD of two to three independent experiments (* *p* < 0.05, ** *p* < 0.01, and *** *p* < 0.001 using one-way ANOVA statistics).

## 3. Results and Discussion

### 3.1. Isolation and Structural Elucidation of Compounds Isolated from P. densiflora 

The cortex and leaves of *P. densiflora* were extracted with MeOH. Following ioassay-guided fractionation, the EtOAc and *n*-BuOH soluble fractions were then fractionated and purified by column chromatography and preparative HPLC, resulting in the isolation of 26 compounds, including two new megastigmane-type compounds (**1** and **2**), 21 diterpenoids (**3**–**23**), and three flavonoids (**24**–**26**). 

Compound **1** was isolated as colorless gum with the molecular formula C_19_H_34_O_7_, which was established from the negatively charged ion [M + HCOO]^−^ at *m/z* 419.2282 (calcd. for C_20_H_35_O_9,_ 419.2287) in the HRESIMS spectrum (Figure S3), corresponding to three double-bond equivalents. The ^1^H NMR data showed the presence of three singlet methyl groups [(*δ*_H_ 1.15 (s, H-11), *δ*_H_ 1.05 (s, H-12) and *δ*_H_ 1.61 (s, H-13)], one doublet methyl group at *δ*_H_ 1.18 (d, *J* = 6.0 Hz, H-10), two oxygenated methine protons [*δ*_H_ 1.61 (dd, *J* = 3.0, 11.0 Hz, H-2) and *δ*_H_ 3.72 (m, H-9)], and sugar ring protons at *δ*_H_ 3.22 ‒ 3.86 (H-2’ ‒ H-5’) along with one anomeric doublet signal at *δ*_H_ 4.35 (d, *J* = 8.0 Hz) (Figure S4). The ^13^C NMR spectrum showed 13 resonances except for the sugar moiety, including two sp^2^ carbons at *δ*_C_ 127.3 and 137.3, two oxygenated sp^3^ carbons at *δ*_C_ 87.9 and 69.2, and nine sp^3^ carbons in the *δ*_C_ 20‒50 ppm range (Figure S5). The characteristic deshielded chemical shifts at *δ*_H_ 3.72 (m)/*δ*_C_ 69.2 of H-9/C-9, the number of carbons of the core structure, and the existence of a long chain (H_7_-H_8_-H_9_-H_10_) and a fragment H_2_-H_3_-H_4_ in the structure supported by the ^1^H-^1^H COSY correlations demonstrated that compound **1** is an ionone derivative of the megastigmane class (Figure S7). As oxygenated C_13_-norisoprenoids, megastigmanes are classified into two series, the damascene series (C-7) and the ionone series (C-9), according to the position of oxygenation. The linkage of the sugar molecule with the aglycone was determined by the HMBC correlation from H-1’ (*δ*_H_ 4.35) to C-2 (*δ*_C_ 87.9) (Figure S6). The planar structure of **1** was also supported by comparison of NMR data with those of the previously reported platanionoside J after hydrolysis, except for the difference in chemical shift of C-2 (*δ*_C_ 87.9 vs. 77.0) where glycosylation occurred in **1** [45].

**Figure 1:**
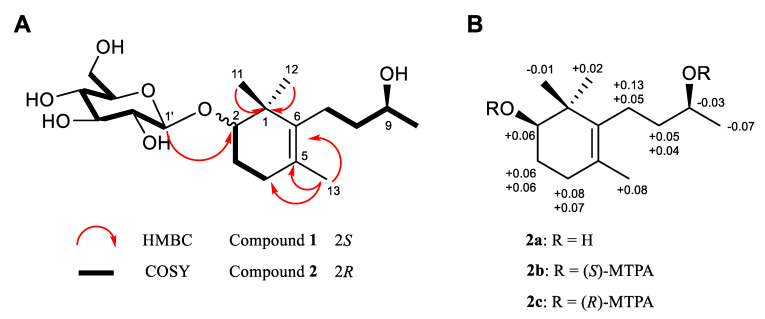
(**A**) Key HMBC (arrows) and COSY (bold) correlations of compounds **1** and **2**. (**B**) Data from the modified Mosher’s method for **2a.**

Compound **2** was also isolated in the same fraction as **1** by preparative HPLC. Its molecular formula was determined to be C_19_H_34_O_7_ by a negative peak [M + HCOO]^−^ at *m/z* 419.2294 (calcd for C_20_H_35_O_9,_ 419.2287) in the HRESIMS spectrum (Figure S8). Interestingly, the ^1^H and ^13^C NMR data of **2** were found to be similar to those of **1** (Table 1), indicating that those two compounds might share the same skeleton but differentiate from each other in absolute configuration. Compound **2** was also determined to be of megastigmane type by four methyl signals at *δ*_H_ 1.14 (s, H-11), 1.04 (s, H-12), 1.64 (s, H-13) and 1.19 (d, *J* = 6.6 Hz, H-10) and 13 carbons constituting the aglycone and sugar moiety, which was observed by an anomeric proton signal resonating at *δ*_H_ 4.36 (d, *J* = 7.8 Hz) and an anomeric carbon at *δ*_C_ 101.8 (Figures S9–S12). By spectral comparison with **1**, the difference in the absolute configuration of compound **2** lies at the C-2 position. The spectral differences originating from C-2 between **1** and **2** were observed in the C-1, 2, 3, and C-1’ positions. Compound **1** exhibited its carbon resonances of C-1, 2, 3 and 1’ at *δ*_C_ 41.5, 87.9, 27.1 and 106.6, respectively, whereas compound **2** showed its corresponding resonances at *δ*_C_ 40.6, 83.0, 23.9, and 101.8, which indicated the possibility of opposite stereochemistry of the C-2 position (Table 1). As the sugar moieties of **1** and **2** were determined to be *β*-d-glucose by ^3^*J*_1’,2’_, which is distinct from that of *α*-d-glucose (Table 1) [46] and the derivatizing method for HPLC analysis (Figure S21) [31], the absolute configuration of C-2 in **1** and **2** could be determined by the application of *β*-d-glucopyranosylation-induced chemical shifts [47]. This can be applied to *β*-d-glucosides of secondary alcohols, especially isoprenoid-*β*-d-glucopyranosides and *β*-d-mannopyranoside, having at least one equatorial alkyl substituent on one of its *β*-carbons. The C-1’ (the first carbon of the sugar moiety) resonance of glucosides of *S*-alcohols is deshielded, appearing at *δ*_C_ 106.4 ± 0.5 ppm, whereas this carbon resonance in an *R*-alcohol appears in a relatively upfield region, *δ*_C_ 101.6 ± 0.9 ppm. Moreover, the signal of the chiral α-carbon where hydroxylation occurs in alcohols is generally displaced by +7.0 ± 0.6 ppm upon glycosylation, whereas a more remarkable deshielding of the α-carbon signal is observed (by approximately +10 ‒ 11 ppm) in the case of *β*-d-glucosides of secondary *S*-alcohols containing at least one equatorial alkyl substituent on one of its *β*-positions. Since the carbon resonances of C-1’ in compounds **1** and **2** appeared at *δ*_C_ 106.6 and 101.8 ppm, respectively, and the chemical shift difference of the C-2, *β*-carbons, between **2** (*δ*_C_ 83.0) and the aglycone of **2** (*δ*_C_ 76.9), the absolute configurations of C-2 in **1** and **2** were readily determined to be *S* and *R*, respectively. Compounds **1** and **2** possess the opposite configuration at C-2 and their NMR spectra were not identical, suggesting that they are in a diastereomeric relationship, and they will have the same stereochemistry at C-9. Therefore, elucidating the absolute configuration of C-9 of either compound will enable the confirmation of C-9 of the other compound. As compound **2** was isolated with a relatively higher yield compared to **1**, compound **2** was subjected to enzymatic hydrolysis and modified Mosher’s analysis [29,30]. As a result (Figure 1, Figures S19 and S20), the stereochemistry at C-9 was determined to be 9*S* and that of C-2 was also confirmed to be 2*R*, initially derived from glycosylation-induced chemical shifts. Hence, compounds **1** and **2** were determined as (2*S*,9*S*)-2,9-dihydroxymegastigman-5-ene-2-*O*-*β*-d-glucopyranoside (**1**) and (2*R*,9*S*)-2,9-dihydroxymegastigman-5-ene-2-*O*-*β*-d-glucopyranoside (**2**).

**Figure 2:**
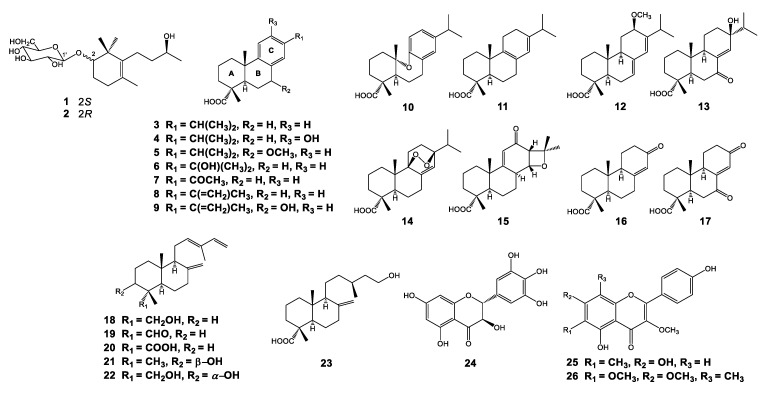
Chemical structures of compounds isolated from *Pinus densiflora*.

The structures of all known compounds isolated from the cortex and leaves of *P. densiflora* were elucidated by extensive analyses of spectroscopic data including NMR and MS spectra and by comparison with previously reported data as follows: (**3**) dehydroabietic acid [48]; (**4**) 12-hydroxydehydroabietic acid [49]; (**5**) 7*α*-methoxydehydroabietic acid [50]; (**6**) 15-hydroxydehydroabietic acid [51]; (**7**) 16-nor-15-oxodehydroabietic acid [52,53]; (**8**) abieta-8,11,13,15-tetraen-18-oic acid [53]; (**9**) 7α-hydroxyabieta-8,11,13,15-tetraen-18-oic acid [54]; (**10**) karamatsuic acid [55]; (**11**) palustric acid [56]; (**12**) 12-methoxy-7,13-abietadien-18-oic acid [57]; (**13**) 7-oxo-13*β*-hydroxyabiet-8(14)-en-18-oic acid [58]; (**14**) 9,13*β*-epidioxy-8(14)-abieten-18-oic acid [59]; (**15**) 14*α*,15-epoxyabiet-9(11)-en-12-oxo-18-oic acid [60]; (**16**) 8(14)-podocarpen-13-on-18-oic acid [51,52]; (**17**) 8(14)-podocarpen-7,13-dion-18-oic acid [51,52]; (**18**) 4-epi-trans-communol [61]; (**19**) 4-epi-trans-communal [62]; (**20**) 4-epi-trans-communic acid [63]; (**21**) 3*β*-hydroxy-12,13(*E*)-biformen [64]; (**22**) 18*α*,3*α*-dihydroxy-12,13(*E*)-biformen [65]; (**23**) (13*S*)-15-hydroxylabd-8(17)-en-18-oic acid [66]; (**24**) ampelopsin [67]; (**25**) 5,7,4′-trihydroxy-3-methoxy-6-*C*-methylflavone [68]; and (**26**) 5,4′-dihydroxy-3,6,7-trimethoxy-8-*C*-methylflavone [69] (see Supplementary Material) (Figure 2).

### 3.2. Influenza Viral Inhibition and Structure-Activity Relationships (SARs) of Isolated Compounds from P. densiflora

**Figure 3:**
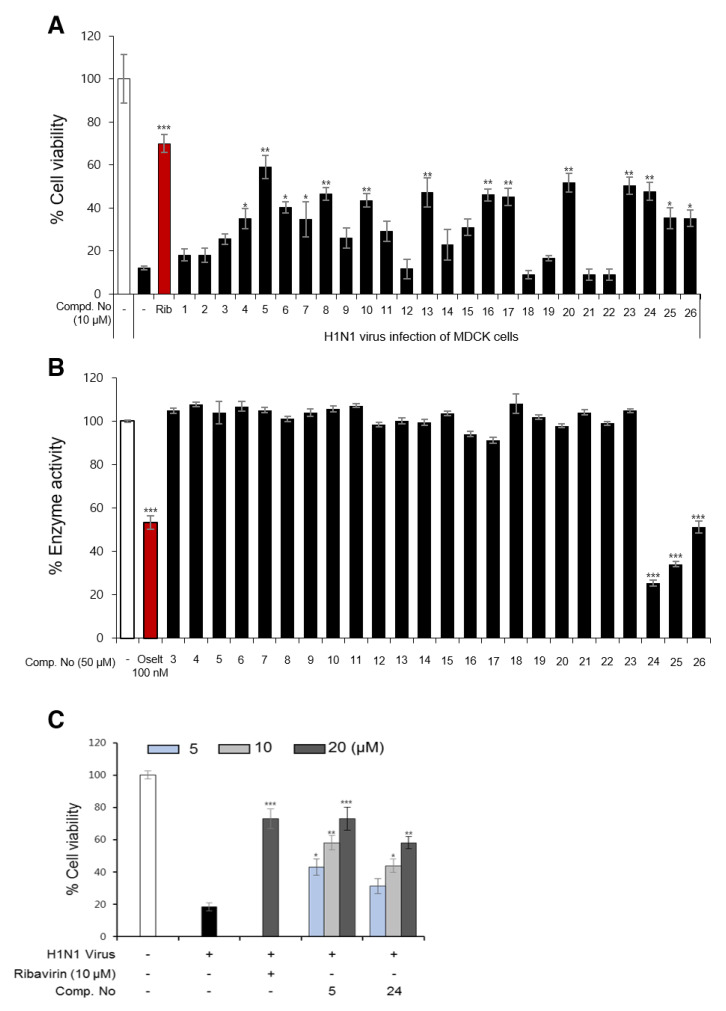
Antiviral activities of isolated compounds against the H1N1 A/PR/8/34 virus in the cytopathic effect (CPE) inhibition and neuraminidase (NA) inhibition assays. (**A**) The percent cell survival was evaluated by the effects of all isolated compounds (**1**–**26**) and ribavirin (Rib), a positive control, at 10 µM with the use of the CPE inhibition assay. (**B**) The inhibitory effects of compounds **3**–**26** at 50 µM and oseltamivir (Oselt; 100 nM), a positive control, on NA from H1N1. (**C**) The influence of compounds **5** and **24** (5, 10, and 20 μM) on cell viability was investigated using the CPE inhibition assay. Values are expressed as the mean ± SD of three independent experiments, * *p* < 0.05, ** *p* < 0.01, and *** *p* < 0.001 compared to the virus control group (one-way analysis of variance followed by a two-tailed Student’s t-test).

The 26 compounds isolated from *P. densiflora* (**1**–**26**) (Figure 1, Figure 2) were screened for their antiviral activities by using CPE inhibition and NA inhibition assays. As shown in Figure 3A and Figure S22, compounds **5**, **6**, **8**, **10**, **13**, **16**, **17**, **20**, **23**, and **24** showed significant inhibition of the cytopathic effect induced by the influenza H1N1 virus at 10 µM with ribavirin as a positive control. Especially, the inhibition activity of compound **5** was comparable to that of ribavirin at the same concentration. Interestingly, nine compounds out of the ten candidates are diterpenoids, and our results are very consistent with the antiviral activity results of some diterpenoids in previously reported papers [70,71,72]. On the other hand, compounds **24–26** were selected as strong candidates from the NA inhibition assay (Figure 3B). The flavonoid substances from *P. densiflora* inhibited NA, and these results can be effectively explained by previously reported studies about the structure–activity relationships (SARs) of compounds in the flavonoid class [73]. Compounds **5** and **24**, which showed the strongest activity from the CPE inhibition assay and NA inhibition assay, respectively, were shown to have CPE inhibitory activity in a dose-dependent manner against the H1N1 and H9N2 subtypes (Figure 3C and Figure S23). From the assessment of effects on cell protection and viral particle neutralization, neither compound protected cells after they were preincubated in the cell protection assay, and they did not neutralize the virulent particles by direct binding (Figure S24). The effect of compound **5** on the cellular apoptosis in infected MDCK cells was also evaluated using flow cytometric analysis. As shown in Figure S27, the percent of apoptotic cells (Sub-G1) significantly decreased from 72.22 to 51.36 and 12.91% after treatment with compound **5** (5 and 20 µM, respectively). These results also suggest that compound **5** has antiviral activity, in part because it inhibits virus-induced cell apoptosis.

To discuss the structure–activity relationships (SARs) of diterpenoids isolated from the *P. densiflora* cortex and leaves, 21 compounds (**3**–**23**) were divided into three groups based on abietane-type tricyclic phenolic (**3**–**10**), tricyclic (**11**–**17**), and labdane-type bicyclic diterpenoids (**18**–**23**). Biological data of group 1 (**3**–**10**) (Figure 3A and Figure S22) indicated that the methoxylation at C-7 (R_2_) could improve the antiviral properties and reduce the cytotoxicity (when comparing compound **5** with **3** and **4**). Compounds **6**–**10**, which replaced the isopropyl group with other groups, still maintained an antiviral effect. However, the presence of the hydroxy moiety at C-7 slightly increased the cytotoxicity (comparing compound **8** with **9**). The antiviral profile of group 2 (**11**–**17**) indicated that the existence of one or two ketone groups at C-7 or C-13 significantly contributed to the anti-influenza activity of the abietane type (comparing compounds **13**, **16**, and **17** with **11**, **12**, **14**, and **15**). The final group (**18**–**23**) demonstrated that substitution of the carboxyl group at C-4 clearly increased H1N1 inhibition. However, replacement of the carboxyl moiety with other groups significantly reduced the antiviral properties, while the cytotoxicity significantly increased (comparing compounds **20** and **23** with **18**, **19** and **21**).

### 3.3. The Effects of Diterpenoids on mRNA and Protein Expression during H1N1 Replication

**Figure 4:**
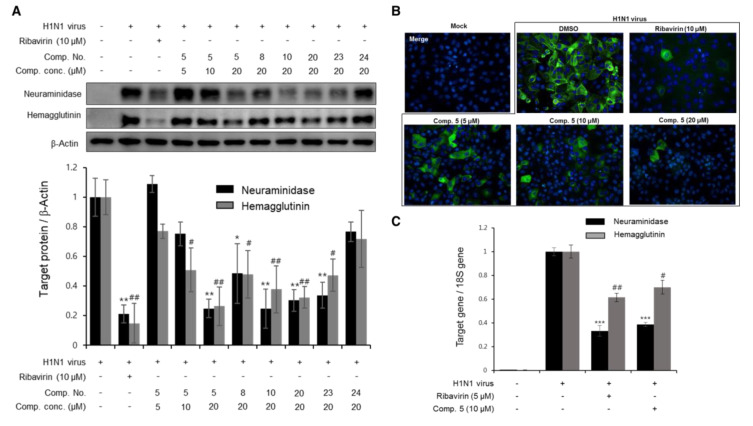
Relative mRNA and protein viral expression levels decreased after treatment with several compounds from *P. densiflora*. (**A**) Madin–Darby Canine Kidney (MDCK) cells were infected with the H1N1 virus for 2 h and then treated with compounds **5**, **8**, **10**, **20**, **23**, and **24** at various concentrations. After 24 h of incubation, viral NA and HA were evaluated by Western blot analysis. Data are presented as the mean ± SD (*n* = 2–4), * *p* < 0.05, ** *p* < 0.01, and # *p* < 0.05, ## *p* < 0.01 compared to the NA and HA virus control groups, respectively. (**B**) Compound **5** reduced NA in the cytoplasm of viral-infected cells as measured by the immunofluorescence method. After infection with the H1N1 virus for 2 h, MDCK cells were incubated with compound **5** or ribavirin as a positive control for 24 h. The cells were then fixed, stained, and visualized using a fluorescence microscope. (**C**) Compound **5** decreased the mRNA levels of NA and HA induced by virus replication in MDCK cells as assessed by real-time PCR. Values are expressed as the mean ± SD (*n* = 3), *** *p* < 0.001, and # *p* < 0.05, ## *p* < 0.01 compared to the NA and HA virus control groups, respectively.

During the viral life cycle, there are several proteins (NA, HA, matrix, NP) that play key roles for replication of the influenza virus. Therefore, inhibition of the synthesis of these proteins could be potentially promising targets for influenza antiviral drug discovery. Based on the CPE inhibition data in Figure 3A, some candidates (compounds **5**, **8**, **10**, **20**, **23** and **24**) were obtained and screened for NA and HA expression by Western blot analysis. After 2 h of infection with the H1N1 virus, the cells were treated with the test compounds at various concentrations with ribavirin as a positive control. The changes in NA and HA expression were analyzed after 24 h of incubation. As shown in Figure 4A, compounds **5**, **8**, **10**, **20** and **23** significantly decreased the protein expression of NA and HA, while compound **24** did not show clear inhibition. Compound **5** was also found to significantly decrease the production of NA and HA in a dose-dependent manner at 5, 10 and 20 μM. These aforementioned results were supported by an immunofluorescence assay (Figure 4B). The green fluorescence of tagged NA was significantly detected in the cell cytoplasm in the viral-infected cells, whereas the fluorescence was not detected in the noninfected cells. Interestingly, when H1N1-infected cells were treated with compound **5** at different concentrations (5, 10, and 20 μM), the green fluorescence similarly decreased in the cell cytoplasm compared to the ribavirin-treated group (10 µM). It was further confirmed by real-time PCR that compound **5** clearly reduced the mRNA expression of NA and HA at the early stage of the viral cycle (Figure 4C).

### 3.4. Detailed Neuraminidase Inhibition Assay of Compound ***24*** against Various Subtypes of Influenza A Virus

**Figure 5:**
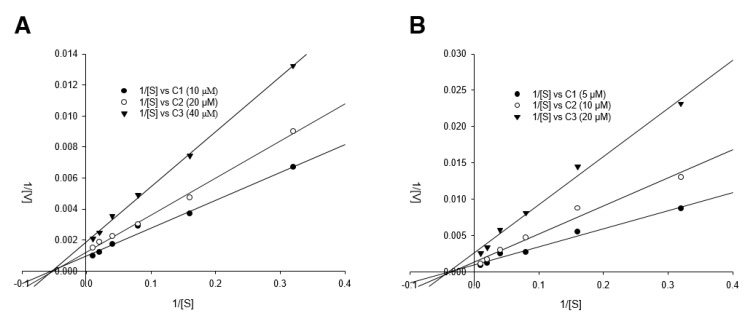
Inhibition mode of compound **24** on the NA H1N1/PR/8/34 and H9N2 A/Chicken/Korea/O1310/2001 enzymes. Lineweaver–Burk plots for the inhibition of compound **24** (**A**) at three concentrations (C1 = 10 μM, C2 = 20 μM, and C3 = 40 μM) on NA from the H1N1 virus and (**B**) at three other concentrations (C1 = 5 μM, C2 = 10 μM, and C3 = 20 μM) on NA from the H9N2 virus for hydrolysis of the substrate. The values were analyzed in three replicates at each substrate concentration.

Based on the screening data of the isolated compounds in Figure 3B, compound **24** was examined for detailed inhibitory NA activity [(including H1N1 A/PR/8/34 virus, H9N2 A/chicken/Korea/01210/2001 virus, wild-type (wt) pandemic 2009 H1N1 virus (A/California/07/2009), oseltamivir-resistant virus (H274Y mutation)]. As shown in Table 2, compound **24** exhibited moderate activity on the percent NA activity, ranging from 35.34 ± 0.97 to 87.33 ± 1.67% in a dose-dependent manner (5, 10, and 20.0 µM). In brief, upon comparison of efficacy of compound **24** with that of oseltamivir, NA activity of H1N1 was inhibited by about 50% by 20 μM of compound **24**, whereas oseltamivir inhibited around 50% at 100 nM. In case of NA of H9N2, NA activity was inhibited around 65% upon treatment of compound **24** at 10 μM, whereas oseltamivir showed around 90% of inhibitory activity even at 100 nM. Similarly, compound **24** showed around 40% enzyme inhibitory activity at 20 μM, whereas 60% of enzymatic activity was reduced by 100 nM of oseltamivir. Based on our previously reported paper [32], the IC_50_ of oseltamivir against NA from H274Y was 7.42 ± 1.35 μM, which is consistent with our present data, but compound **24** inhibited around 53% of inhibitory activity at 20 μM. Notably, the inhibitory activity of oseltamivir (100 nM) against the oseltamivir-resistant novel H1N1 (H275Y mutant) decreased greatly compared to the activity against H1N1 A/PR/8/34 and novel H1N1 (wt) (from 49.90 ± 3.74 and 38.57 ± 4.32 to 91.10 ± 4.07%, respectively), whereas compound **24** retained its inhibitory activity even against the oseltamivir-resistant novel H1N1 (from 48.40 ± 1.35 and 58.76 ± 1.85 to 57.22 ± 1.11%, respectively). Compound **24** was further assessed for its inhibitory mode against NA from H1N1 and H9N2 using a double-reciprocal Lineweaver–Burk plot. The result in Figure 5 shows that compound **24** was found to be a noncompetitive inhibitor in both enzyme subtypes (H1N1 and H9N2), because raising the concentration of substrate resulted in the convergence of lines that intersected at a nonzero point on the negative x-axis. Thus, compound **24** may allosterically interrupt NA activity that cleaves the binding between the sialic acid residues of the receptor of cells and a newly formed virion [74].

### 3.5. Molecular Docking Simulation of Compounds ***5*** and ***24*** with Expected Target Proteins

**Figure 6:**
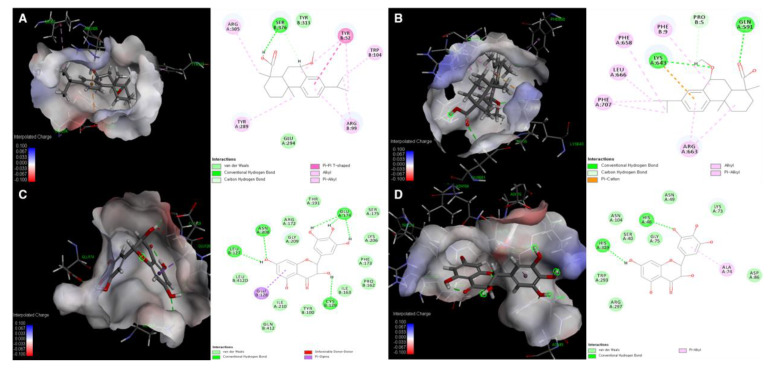
Illustration of the molecular docking simulation and 2D diagram of the interactions between (**A**) NP and compound **5**, (**B**) PA-PB1 and compound **5**, (**C**) NA and compound **24**, and (**D**) NA mutant and compound **24** using Discovery Studio Client v19.1.0.18287/CDOCKER software. The structures of the proteins were obtained from the Protein Data Bank (http://www.pdb.org) (PDB ID code: 3RO5, 2ZNL, 3TI4, and 3CL0 for A-D, respectively).

From the results in Figure 4, it is evident that compound **5** inhibits the synthesis of viral membrane proteins, HA and NA, but it is not obvious how this inhibition occurs. Therefore, molecular docking (MD) analysis was performed to predict which steps are disturbed for the inhibition of synthesis of HA and NA through binding affinity calculation and this MD analysis will serve as reference data for the future mechanistic in vitro studies. Since there are numerous target points in cascade reaction for the synthesis of two proteins and there become more relevant factors as the cascades proceeds further, it is advantageous to try first MD simulation with proteins in the upstream of the cascade, such as polymerase subunits and NP which are responsible for viral protein synthesis. Polymerase proteins including polymerase acidic protein (PA), polymerase basic protein 1 and 2 (PB1 and PB2), and NP, structural proteins of RNP particles play important roles in the life cycle of the influenza virus, such as during replication and transcription. NP is especially involved in RNP trafficking between the nucleus and cytoplasm and in the replication of RNPs and directly binds with M1, PB1, and PB2 [75,76]. Therefore, without NP, synthesis of all the viral proteins including HA and NA are ultimately prohibited via inhibition of mRNA transcription and vRNA replication. Moreover, previous studies have reported that the maximum amino acid difference of NP among influenza A strains was less than 11%, which makes NP as a potential therapeutic target for the treatment of all influenza virus strains [77]. In addition to NP, after polymerase subunits (PA, PB1, and PB2) are newly synthesized via initial mRNA expression and imported back into nucleus, each subunit plays its own distinct roles in mRNA/vRNA transcription. PB1 subunit serves as a place where complementary RNA (cRNA) is elongated [78]. Also, in the cap snatching step, which is a crucial for mRNA transcription, PB2 and PA are used for binding 5’ cap of a host mRNA and cleaving nucleotides of the 5’ cap, respectively [79,80]. Considering the results in Figure 4 and importance of NA and polymerase subunits in terms of synthesis of HA and NA, we investigated whether compound **5** can be docked to polymerase proteins and NP of influenza virus. According to the data in the Protein Data Bank (PDB), the binding pocket between the active ligand 4-(2-chloro-4-nitrophenyl)piperazin-1-yl][3-(2-methoxyphenyl)-5-methyl-1,2-oxazol-4-yl]methanone (LGH) and NP (PDB ID: 3RO5) showed stable interaction energy at residues Arg305, Ser376, Tyr52, Tyr289, and Arg99 [41] (Figure 6). In our study, compound **5** showed several interactions with Arg305, Ser376, Tyr52, Trp104, Arg99, Tyr289, Typ313, and Glu294 in active site AC1 with a low CDOCKER energy and CDOCKER interaction energy (−17.2458 and −40.9050 kcal/mol, respectively; Tables S1 and S2).

In addition to the NP complex, docking analysis of **5** with the structures of the C-terminal region of PA (PAC) complexed with a peptide from PB1 was carried out. A previous report suggested that residues Met1, Val3, Asn4, Pro5, Leu7, Leu8, Phe9, and Leu10 on the PB1 peptide are important key residues for interactions with PA and show the lowest binding free energy [42]. In order to determine the mechanism of binding of **5** with PA-PB1 polymerase, the interactions of compound **5** and the polymerase complex (PDB ID: 2ZNL) were identified at residues Pro5, Phe9, Lys643, Gln591, Phe658, Phe707, Leu666, and Arg663 with a low CDOCKER energy and CDOCKER interaction energy (−7.5123 and −32.6849 kcal/mol, respectively; Figure 6 and Tables S1 and S2). When comparing CDOCKER energy values of two docking enzymes of compound **5**, it is likely that compound **5** binds with NP in a more stable manner than with PA-PB1.

Based on the data in Figure 3B and Table 2, in which compound **24** showed strong NA inhibitory activity against four types of IVA, docking analysis of **24** with NA (PDB ID: 3TI4) [43] and its mutant (PDB ID: 3CL0) [44] was implemented. Since results in Figure 5 clearly indicated that compound **24** inhibits neuraminidase in a noncompetitive manner, in other words, it binds to allosteric sites, docking sites were obtained by defining binding sites from receptor cavities of whole structures of the enzyme to perform blind docking experiments. As shown in Figure 6, Figure S28 and Tables S1–S3, compound **24** showed stable docking with nine binding sites out of 21 sites. Among the nine binding sites, compound **24** bound to site 1 with the lowest CDOCKER energy (−38.5234 kcal/mol), which means its binding is fairly stable compared to the value of docking of laninamivir octanoate (positive control). Although interaction amino acid residues from both cases cannot be compared with each other since laninamivir octanoate inhibits the NA in a competitive manner, only CDOCKER energy and CDOCKER interaction energy could be compared to see how the docking of our compound **24** is stable. It binds to Glu174, Asn208, Leu127, and Cys129 via conventional hydrogen bonds and with Glu128 via pi-sigma interaction (Figure 6). On the other hand, compound **24** was stably docked to four binding sites of NA mutant out of seven sites (Figure 6 and Tables S1, S2, and S4). Among them, the compound was successfully docked to site 6 with the lowest energy of CDOCKER energy (−24.4057 kcal/mol) compared to that of docking of oseltamivir (Figure S28). It interacts with His46 and His329 via a conventional hydrogen bond and has pi-alkyl interaction with Ala74 residue of the enzyme (Figure 6). Taken together, the MD analysis of compound **5** with NP and PA-PB1 suggests that it may have inhibitory influences on NP rather than on PA-PB1 and serves as reference for the future in vitro assay to verify. Also, the result of MD analysis of compound **24** supports its strong inhibitory activity toward NA and NA mutant and suggests the possible allosteric interaction residues of enzymes.

### 3.6. Anti-Inflammatory Effects of the Isolated Compounds

**Figure 7:**
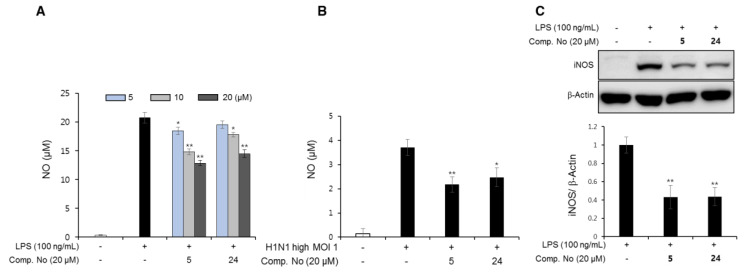
Effects of compounds **5** and **24** on NO production and iNOS expression in RAW 264.7 cells. Treatment of the test compounds at various concentrations to inhibit NO production in RAW 264.7 cells by either (**A**) 100 ng/mL LPS treatment or (**B**) H1N1 A/PR/8/34 viral infection. (**C**) Western blot analysis of iNOS expression in activated RAW 264.7 cells decreased by compounds **5** and **24** at 20 μM. Data are presented as the mean ± SD (*n* = 3), * *p* < 0.05, ** *p* < 0.01, compared to the positive control group.

Combination chemotherapy between NA inhibitors and anti-inflammatory agents may become a new strategy against drug resistance in patients with influenza viral infections. Previous studies have reported that during influenza viral infections, proinflammatory cytokines and nitric oxide (NO) production could be excessive in macrophages. In this study, we evaluated the ability of isolated compounds from *P. densiflora* to suppress the production of NO and inducible nitric oxide synthase (iNOS) expression in RAW cells. As shown in Figure 7A and Figure S25, compounds **5** and **24** did not show cytotoxicity at 30 μM and inhibited NO production in a dose-dependent manner at 5, 10, and 20 μM in RAW cells stimulated with 100 ng/mL LPS. This tendency was reproduced in RAW cells upon viral infection. In our preliminary experiments with low MOI 0.01 infections of the H1N1 A/PR/8/34 virus, NO production was low and inconsistent (Figure S26). Therefore, infections with a high MOI 1.0 were implemented. As shown in Figure 7B, with a high MOI 1 infection, NO production was 11-fold higher than that in the low MOI 0.01 infections. Moreover, NO production was clearly reduced in the presence of compounds **5** and **24** (20 μM). Moreover, the expression of iNOS significantly decreased when stimulated RAW cells were treated with these compounds at the concentration of 20 µM (Figure 7C). Based on the above data, we gained insight into the effects of the two compounds on the inhibition of NO production. Considering the high mortality induced by complications such as secondary bacterial pneumonia and otitis media following influenza infection, prevention of excessive inflammatory responses is also important. It has been demonstrated that severe IAV infection can give rise to pulmonary infiltration and hypoxemia that ultimately leads to acute respiratory distress syndrome (ARDS), the major contributor to severe mortality [81]. Pathophysiologically, it has been shown that inflammation upon viral infection occurs to adapt to the body by increasing body temperature and recruiting monocytes/macrophages in order to resist and fight against invasion as a normal defensive mechanism. However, it is possible that the immune system of our body releases excessive levels of proinflammatory cytokines and chemokines. A strong Th1-type immune response stimulated by proinflammatory cytokines, such as TNF-*α* and IL-1*β*, causes tissue damage [82,83]. Although detailed experiments, such as the effects on the aforementioned cytokines in lung or spleen tissue, are needed, our results indicated that compounds **5** and **24** from *P. densiflora* can effectively inhibit not only viral growth in MDCK cells but also NO production by multitargeting.

## 4. Conclusions

Taken together, 26 compounds, including two new megastigmane glycosides, 23 diterpenoids, and three flavonoids, were isolated from the cortex and leaves of *P. densiflora*. Whole compounds were evaluated for their CPE and NA inhibitory properties, which in turn allowed us to anticipate SAR information. Some candidates that showed potent activity were selected and subjected to various detailed mechanistic studies to identify the mode of action, such as effects on mRNA expression, cellular apoptosis, neutralization of viral particles, cell protection against viral infection and inhibition of inflammation. While flavonoids exerted their anti-influenza activity via direct NA inhibition, the diterpenes isolated here are likely to affect the gene expression of various proteins that are essential for viral propagation rather than direct neutralization of surface proteins. Moreover, considering that compound **5** exhibited anti-inflammatory activity, other isolated diterpenes may also have that activity. To date, this study demonstrates that *P. densiflora* can be considered an abundant natural anti-influenza viral source to find new bioactive compounds against influenza virus outbreaks in the future.

## Figures and Tables

**Table 1:** NMR Spectroscopic data for compounds **1** and **2.**

No.	1*^a^*		2*^a^*	
	*δ*_H_ (*J* in Hz)	*δ*_C_	*δ*_H_ (*J* in Hz)	*δ*_C_
**1**		41.5		40.6
**2**	3.43 dd (3.0, 11.0)	87.9	3.62 dd (3.0, 10.2)	83.0
**3**	2.04 m	27.1	1.86 m	23.9
	1.79 m		1.74 m	
**4**	2.05 m	31.7	2.11 td (4.8, 16.8)	31.3
			2.02 m	
**5**		127.3		127.1
**6**		137.3		137.5
**7**	2.15 dt (4.5, 13.0)	26.0	2.21 dt (4.8, 12.0)	26.1
	2.00 m		1.98 m	
**8**	1.51 m	40.7	1.53 m	40.8
**9**	3.72 m	69.2	3.73 m	69.2
**10**	1.18 d (6.0)	23.3	1.19 d (6.6)	23.3
**11**	1.15 s	22.5	1.14 s	22.7
**12**	1.05 s	26.4	1.04 s	26.7
**13**	1.61 s	19.7	1.64 s	19.7
**Glu1**	4.35 d (8.0)	106.6	4.36 d (7.8)	101.8
**Glu2**	3.22 t (8.0)	75.7	3.22 dd (7.8, 9.0)	75.1
**Glu3**	3.36 m	78.3	3.39 t (9.0)	78.3
**Glu4**	3.28 m	71.7	3.31 t (9.0)	71.9
**Glu5**	3.26 m	77.7	3.26 m	77.8
**Glu6**	3.86 dd (2.0, 11.5)	62.8	3.88 dd (1.8, 11.4)	63.0
	3.68 dd (5.5, 11.5)		3.69 dd (6.0, 11.4)	

*^a^* Recorded in methanol-*d*_4_ at 500 MHz.

**Table 2:** Percent influenza NA activity (compared with DMSO as a control).

Comp. Name	H1N1	H9N2	H1N1 (wt)	H274Y
DMSO	100 ± 2.69	100 ± 0.62	100 ± 1.04	100 ± 6.51
Oseltamivir (100 nM)	49.90 ± 3.74	10.79 ± 0.18	38.57 ± 4.32	91.10 ± 4.07
Comp. **24** (5 μM)	75.93 ± 1.17	64.19 ± 1.92	87.33 ± 1.67	76.73 ± 1.59
Comp. **24** (10 μM)	62.55 ± 0.94	49.79 ± 1.74	76.23 ± 2.67	72.38 ± 6.96
Comp. **24** (20 μM)	48.40 ± 1.35	35.34 ± 0.97	58.76 ± 1.85	57.22 ± 1.11

## Supplementary Materials

The following are available online at https://www.mdpi.com/2218-273X/10/5/711/s1. Figure S1: Isolation scheme of the compounds from the leaves of *Pinus densiflora.* Figure S2: Isolation scheme of the compounds from the cotex of *Pinus densiflora.* Figure S3: HRESIMS data of compound **1**. Figure S4: ^1^H NMR spectrum (methanol-*d_4_*, 500 MHz) of compound **1**. Figure S5: ^13^C NMR spectrum (methanol-*d_4_*, 125 MHz) of compound **1**. Figure S6: HMBC spectrum (methanol-*d_4_*, 500 MHz) of compound **1**. Figure S7: COSY spectrum (methanol-*d_4_*, 500 MHz) of compound **1**. Figure S8: HRESIMS data of compound **2**. Figure S9: ^1^H NMR spectrum (methanol-*d_4_*, 500 MHz) of compound **2**. Figure S10: ^13^C NMR spectrum (methanol-*d_4_*, 125 MHz) of compound **2**. Figure S11: HMBC spectrum (methanol-*d_4_*, 500 MHz) of compound **2**. Figure S12: COSY spectrum (methanol-*d_4_*, 500 MHz) of compound **2**. Figure S13: HRESIMS data of compound **2a**. Figure S14: ^1^H NMR spectrum (methanol-*d_4_*, 400 MHz) of compound **2a.** Figure S15: ^13^C NMR spectrum (methanol-*d_4_*, 100 MHz) of compound **2a**. Figure S16: HSQC NMR spectrum (methanol-*d_4_*, 400 MHz) of compound **2a**. Figure S17: HRESIMS data of compound **2b**. Figure S18: HRESIMS data of compound **2c**. Figure S19: ^1^H NMR spectrum (CDCl_3_, 600 MHz) of compound **2b** and **2c**. Figure S20: COSY spectrum (CDCl_3_, 600 MHz) of compound **2b** and **2c**. Figure S21: Comparison of chromatograms after sugar analysis for the determination of absolute configuration of sugar moiety in compound **1** and **2**. Figure S22: Percent cell viability evaluated by the effects of all compounds (**1**–**26**) at 10 µM using cytotoxicity assay. Figure S23: Inhibition of cytopathic effect of compounds **5** and **24** against H9N2 virus. Figure S24: The effects of compounds **5** and **24** on the cell protection from viral infection and the H1N1 particles. Figure S25: Percent cell viability of RAW 264.7 cells evaluated by the effects of compounds **5** and **24** at 30 µM using cytotoxicity assay. Figure S26: Effects of compounds **5** and **24** on the NO production in infected-RAW 264.7 cells with low MOI. Figure S27: The effects of compound **5** on the cellular DNA contents after H1N1 virus infection. Figure S28: Images of global representation of NA surface with selected docking sites for compound **24**. Table S1: CDOCKER and CDOCKER interaction energies of compounds **5** and **24** with nucleoprotein, PA-PB1 polymerase, neuraminidase, and neuraminidase mutant. Table S2: Molecular docking and interaction images of positive control with nucleoprotein, neuraminidase, and neuraminidase mutant. Table S3: CDOCKER and CDOCKER interaction energies of compounds **24** with neuraminidase. Table S4: CDOCKER and CDOCKER interaction energies of compounds **24** with neuraminidase mutant. Table S5: Primers used for real-time PCR. Supporting NMR data of isolated compounds.
