# Supplementary material for: Antiviral Activities of Compounds Isolated from Pinus densiflora (Pine Tree) against the Influenza A Virus
Source: Biomolecules. 2020 May 4;10(5):711. doi: 10.3390/biom10050711 (PMC7278015; doi:10.3390/biom10050711)
Supplement: Supplementary file 1 [file biomolecules-10-00711-s001.docx]

Article

Antiviral Activities of Compounds Isolated from *Pinus densiflora* (Pine Tree) against the Influenza A Virus

Thi Kim Quy Ha ^1,2,†^, Ba Wool Lee ^1,†^, Ngoc Hieu Nguyen ^1^, Hyo Moon Cho ^1^, Thamizhiniyan Venkatesan ^1^, Thi Phuong Doan ^1^, Eunhee Kim ^3^ and Won Keun Oh ^1,^*

^1^ Korea Bioactive Natural Material Bank, Research Institute of Pharmaceutical Sciences, College of Pharmacy, Seoul National University, Seoul 08826, Republic of Korea; htkquy@ctu.edu.vn (T.K.Q.H.); paul36@snu.ac.kr (B.W.L.); hieusnu@gmail.com (N.H.N.); chgyand@naver.com (H.M.C.); thamtris@snu.ac.kr (T.V.); phuongdoan@snu.ac.kr (T.P.D.); wkoh1@snu.ac.kr (W.K.O.)

^2^ College of Natural Sciences, Cantho University, Campus II, Cantho City, Vietnam

^3^ Choong Ang Vaccine Laboratory, 1476-37, Yuseong-daero, Yuseong-gu, Daejeon 34055, Republic of Korea; ehkim@cavac.co.kr

***** Correspondence: wkoh1@snu.ac.kr; Tel.: +82-2-880-7872

† These authors contributed equally to this work

**Contents:**

**Fig. S1:** Isolation scheme of the compounds from the leaves of *Pinus densiflora*

**Fig. S2:** Isolation scheme of the compounds from the cotex of *Pinus densiflora*

**Fig. S3:** HRESIMS data of compound **1**

**Fig. S4**: ^1^H NMR spectrum (methanol-*d_4_*, 500 MHz) of compound **1**

**Fig. S5**: ^13^C NMR spectrum (methanol-*d_4_*, 125 MHz) of compound **1**

**Fig. S6**: HMBC spectrum (methanol-*d_4_*, 500 MHz) of compound **1**

**Fig. S7**: COSY spectrum (methanol-*d_4_*, 500 MHz) of compound **1**

**Fig. S8:** HRESIMS data of compound **2**

**Fig. S9**: ^1^H NMR spectrum (methanol-*d_4_*, 500 MHz) of compound **2**

**Fig. S10**: ^13^C NMR spectrum (methanol-*d_4_*, 125 MHz) of compound **2**

**Fig. S11**: HMBC spectrum (methanol-*d_4_*, 500 MHz) of compound **2**

**Fig. S12**: COSY spectrum (methanol-*d_4_*, 500 MHz) of compound **2**

**Fig. S13:** HRESIMS data of compound **2a**

**Fig. S14**: ^1^H NMR spectrum (methanol-*d_4_*, 400 MHz) of compound **2a**

**Fig. S15**: ^13^C NMR spectrum (methanol-*d_4_*, 100 MHz) of compound **2a**

**Fig. S16**: HSQC NMR spectrum (methanol-*d_4_*, 400 MHz) of compound **2a**

**Fig. S17:** HRESIMS data of compound **2b**

**Fig. S18:** HRESIMS data of compound **2c**

**Fig. S19**: ^1^H NMR spectrum (CDCl_3_, 600 MHz) of compound **2b** and **2c**

**Fig. S20**: COSY spectrum (CDCl_3_, 600 MHz) of compound **2b** and **2c**

**Fig. S21:** Comparison of chromatograms after sugar analysis for the determination of absolute

configuration of sugar moiety in compound **1** and **2**.

**Fig. S22:** Percent cell viability evaluated by the effects of all compounds (**1**−**26**) at 10 *µ*M using

cytotoxicity assay. MDCK cells were incubated with test compounds for 2 days. MTT assay was

carried out and measured at wavelength 550 nm. Values were expressed as mean ± SD of three

independent experiments, **p* < 0.05, ***p* < 0.01, compared to the control group.

**Fig. S23**: Inhibition of cytopathic effect of compounds **5** and **24** against H9N2 virus. MDCK cells

were infected with H9N2 virus for 2 h, and then the cells were treated with compounds **5** and

**24** at different concentrations. After 3 days of incubation, the antiviral activity was evaluated

using CPE inhibition assay. Values are expressed as the mean ± SD (*n* = 3), **p* < 0.05, ***p* < 0.01,

*** *p* < 0.001, as compared to the virus control group.

**Fig. S24:** The effects of compounds **5** and **24** on the cell protection from viral infection and the

H1N1 particles. Briefly, for cell protection assay for H1N1 infection, MDCK cells were pre-

incubated with compounds for 4 h before the H1N1 A/PR/8/34 virus absorbed into the cells.

After 2 h incubation, the culture was washed with PBS and replaced by new media and

incubated for 3 days. Antiviral activity was determined by a cytotoxicity assay. Similarly, the

infectivity of H1N1 particles assay was carried out with or without pre-incubation of compounds

and H1N1 virus for 1 h at 4 °C. Then, these viral media were absorbed into MDCK cells for 1 h

at 37 °C under 5% CO_2_ atmosphere. The cells were washed two times with PBS and incubated

for 3 days in new media. Then, antiviral activity was determined by cytotoxicity assay.

**Fig. S25:** Percent cell viability evaluated by the effects of compounds **5** and **24** at 30 *µ*M using

cytotoxicity assay. RAW 264.7 cells were incubated with test compounds for 1 days. MTT assay

was carried out and measured at wavelength 550 nm. Values were expressed as mean ± SD of

three independent experiments, **p* < 0.05, ***p* < 0.01, compared to the control group.

**Fig. S26:** Effects of compounds **5** and **24** on the NO production in infected-RAW 264.7 cells with

low MOI. The cells were exposed to compounds for 10 h, followed by the infection with the

H1N1 A/PR/8/34 virus of low MOI 0.01 for 1 h. After 12 h of incubation, NO production was

measured using the Griess reagent method.

**Fig. S27:** The effects of compound **5** on the cellular DNA contents 3 days after H1N1 virus

infection. The flow cytometric analysis was performed to detect the percentage of apoptotic cells

(Sub-G1) using histograms for the viral-infected cell cycle.

**Fig. S28:** Images of global representation of NA surface (A: NA from H1N1 (wt), B: NA from

H1N1 mutant) with selected docking sites where compound **24** was docked successfully with

stable CDOCKER energy (below 0 kcal/mol)

**Table S1:** CDOCKER and CDOCKER interaction energies of compounds **5** and **24** with

nucleoprotein, PA-PB1 polymerase, neuraminidase, and neuraminidase mutant.

**Table S2:** Molecular docking and interaction images of positive control with nucleoprotein,

neuraminidase, and neuraminidase mutant.

**Table S3:** CDOCKER and CDOCKER interaction energies of compounds **24** with neuraminidase.

**Table S4:** CDOCKER and CDOCKER interaction energies of compounds **24** with neuraminidase

mutant.

**Table S5:** Primers used for Real-time PCR.

**Supporting NMR data:** ^1^H NMR and ^13^C NMR of all isolated-compounds from the leaves and

cotex of *Pinus densiflora*.


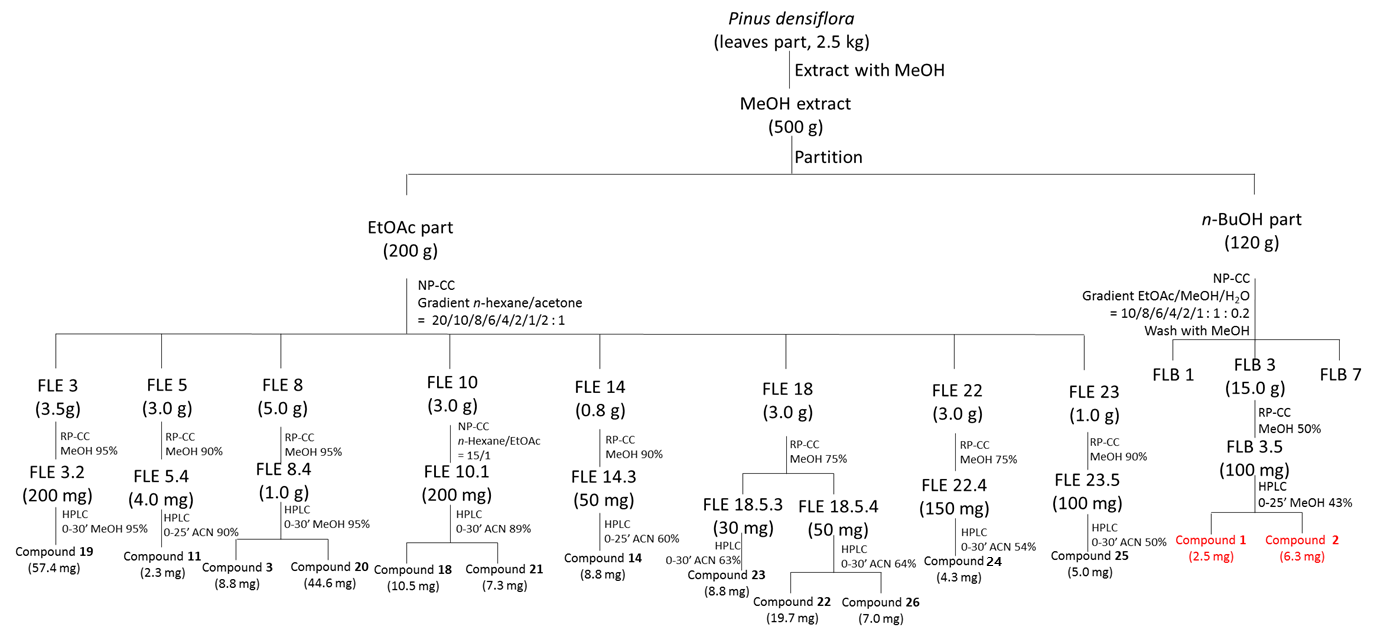


**Figure S1.** Isolation scheme of the compounds from the leaves of *Pinus densiflora.*


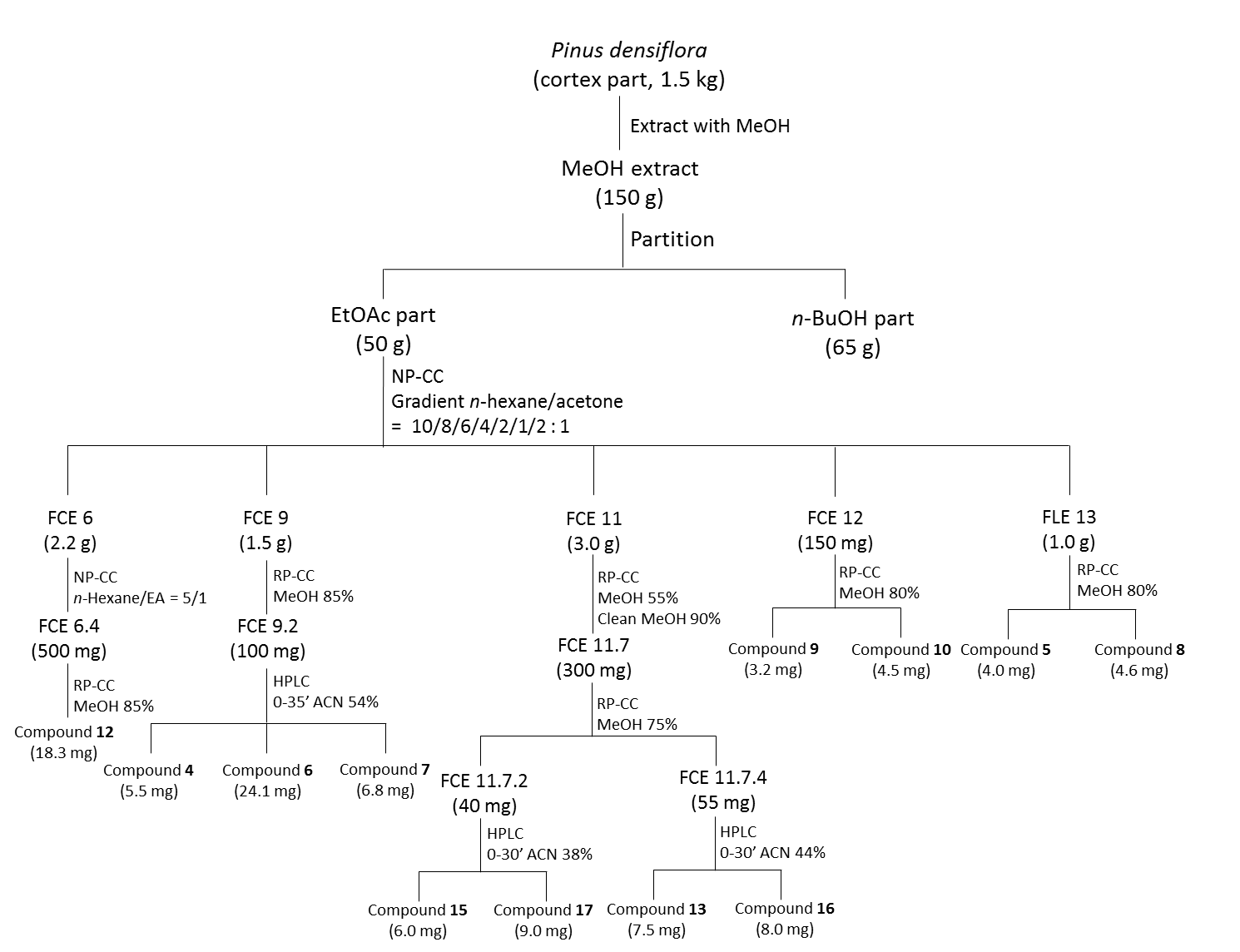


**Figure S2.** Isolation scheme of the compounds from the cotex of *Pinus densiflora.*


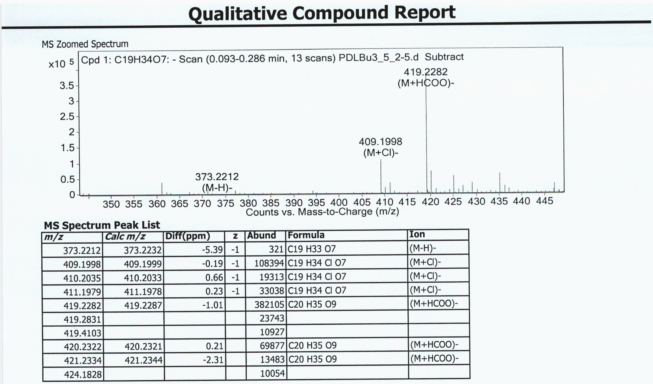


**Figure S3.** HRESIMS data of compound **1.**


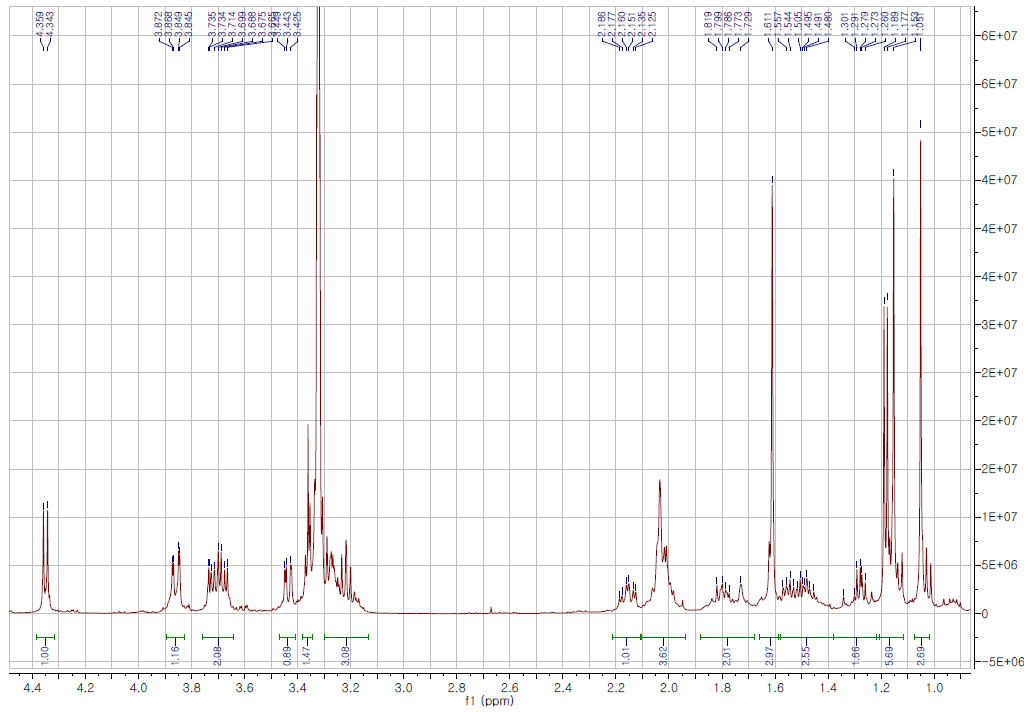


**Figure S4.** ^1^H NMR spectrum (methanol-*d_4_*, 500 MHz) of compound **1.**


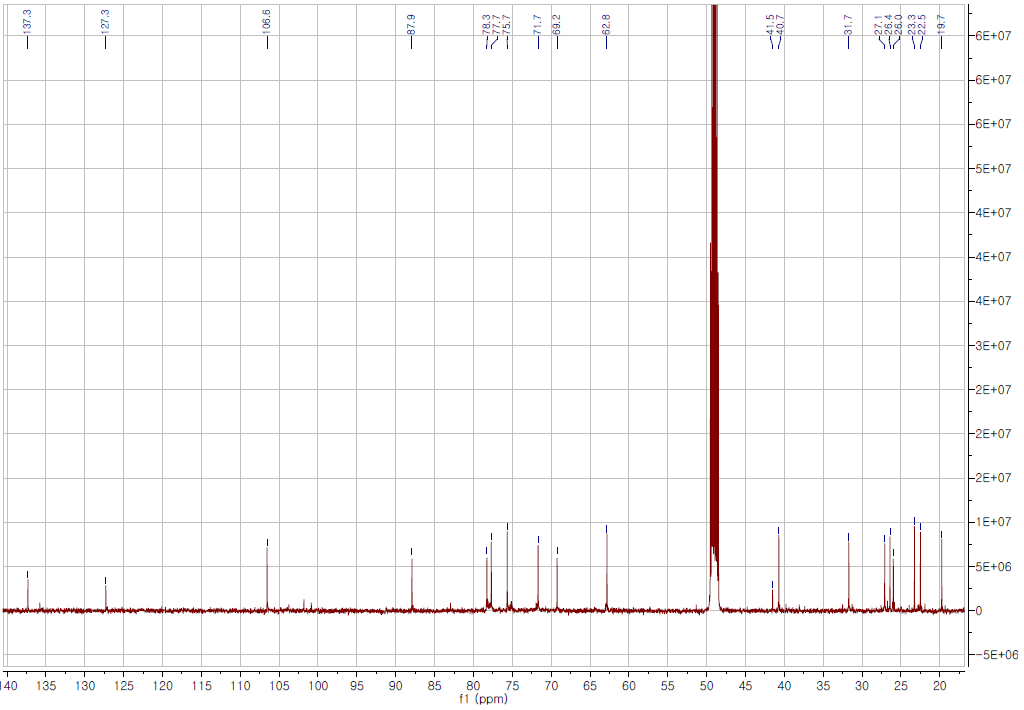


**Figure S5.** ^13^C NMR spectrum (methanol-*d_4_*, 125 MHz) of compound **1.**


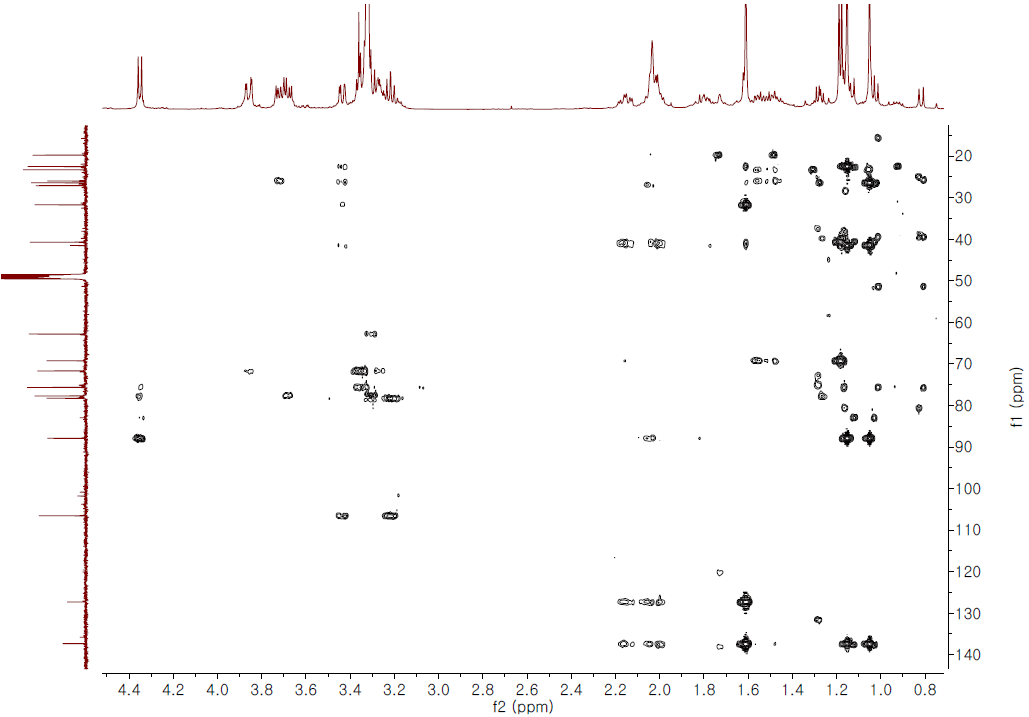


**Figure S6.** HMBC spectrum (methanol-*d_4_*, 500 MHz) of compound **1.**


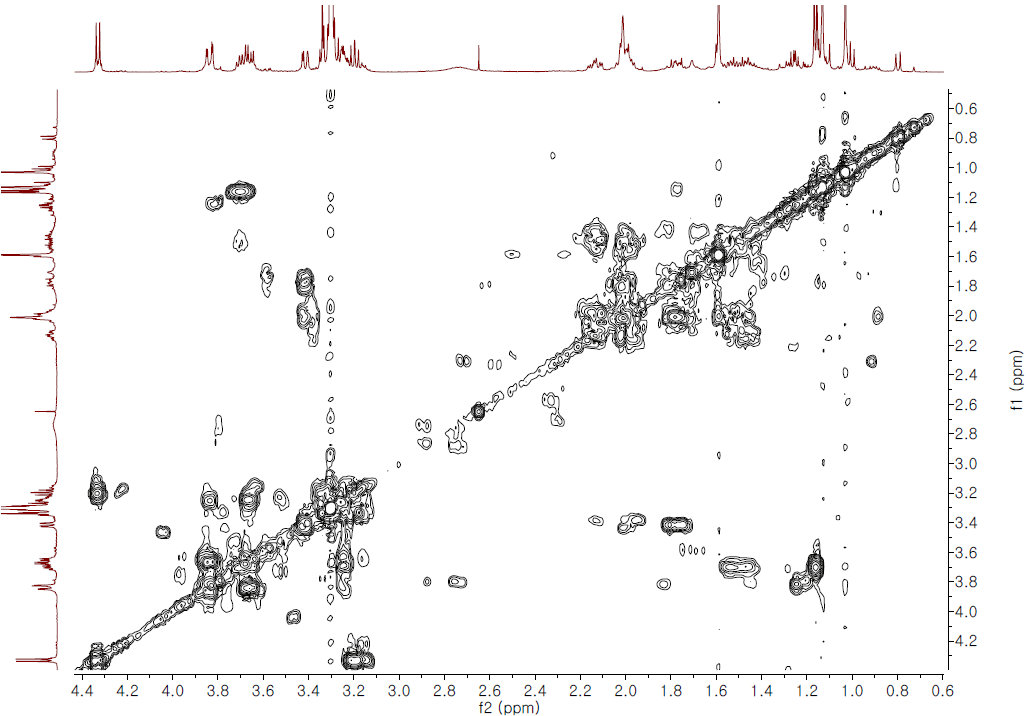


**Figure S7.** COSY spectrum (methanol-*d_4_*, 500 MHz) of compound **1.**


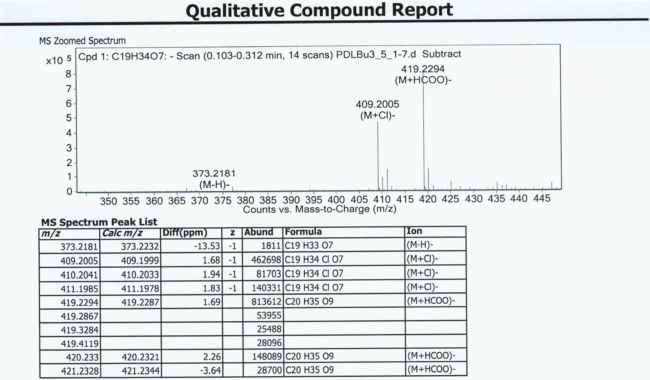


**Figure S8.** HRESIMS data of compound **2.**


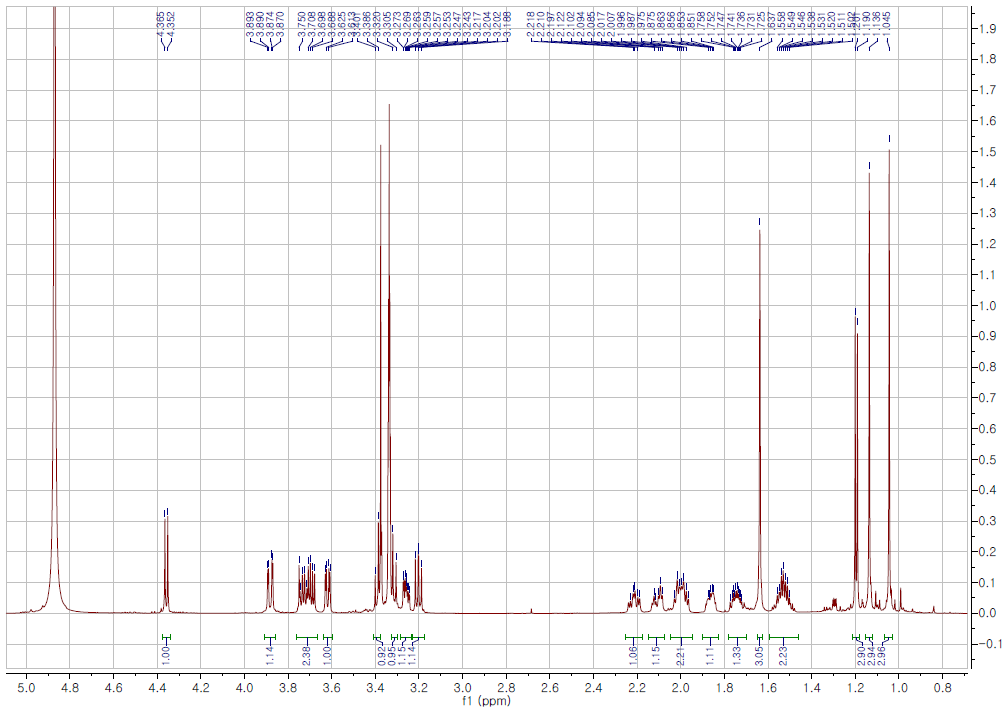


**Figure S9.** ^1^H NMR spectrum (methanol-*d_4_*, 500 MHz) of compound **2.**


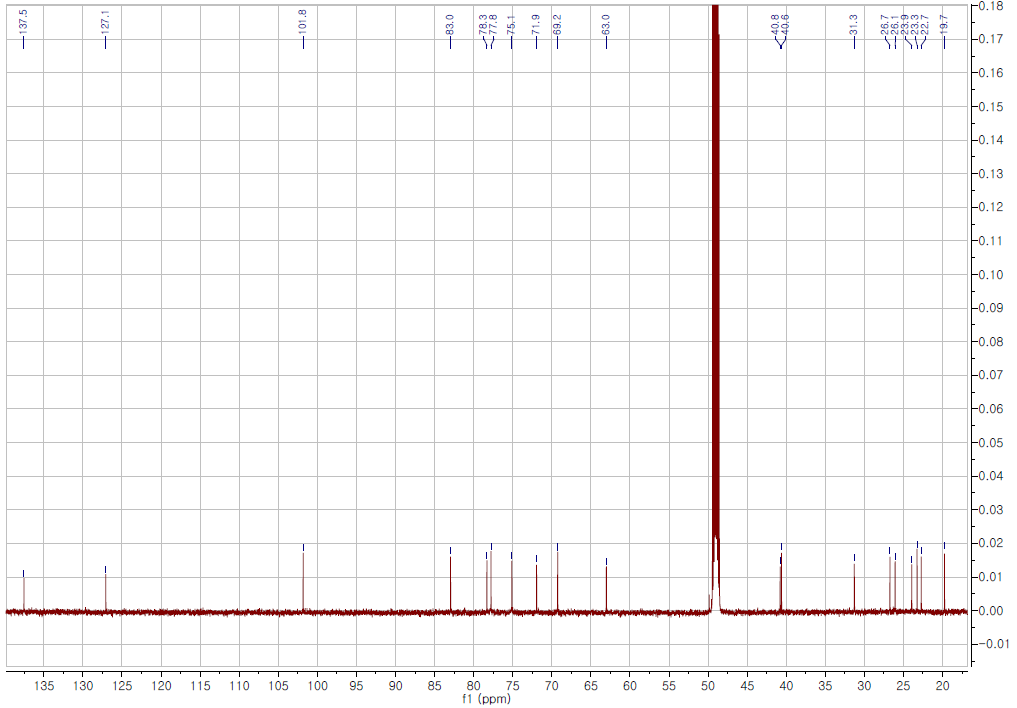


**Figure S10.** ^13^C NMR spectrum (methanol-*d_4_*, 125 MHz) of compound **2.**


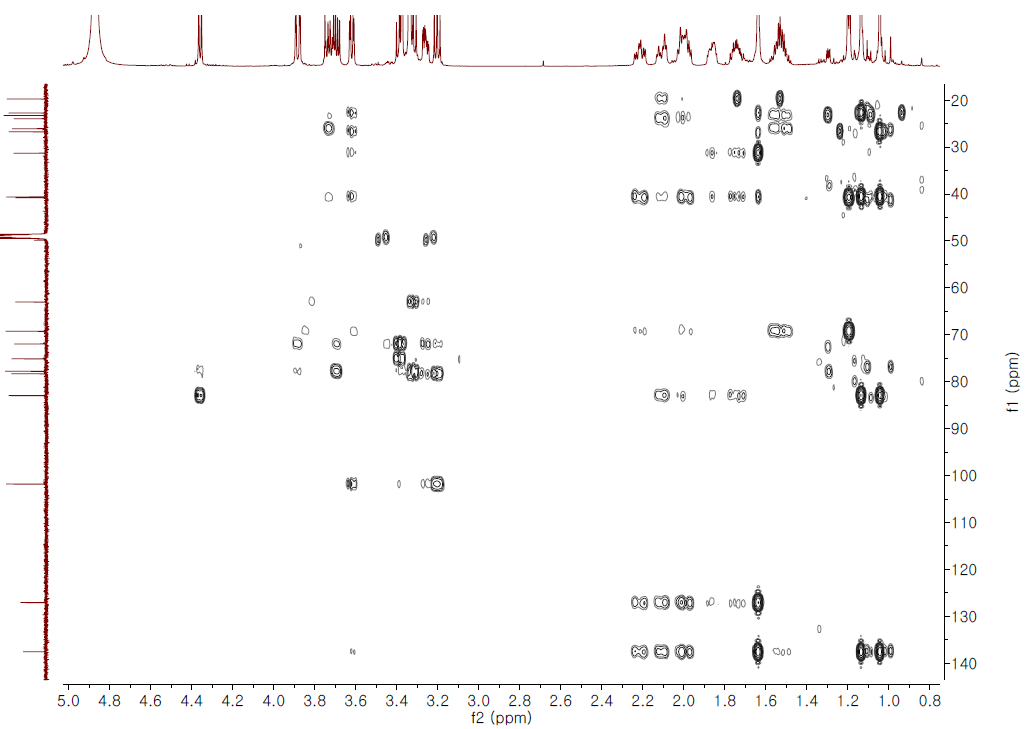


**Figure S11.** HMBC spectrum (methanol-*d_4_*, 500 MHz) of compound **2.**


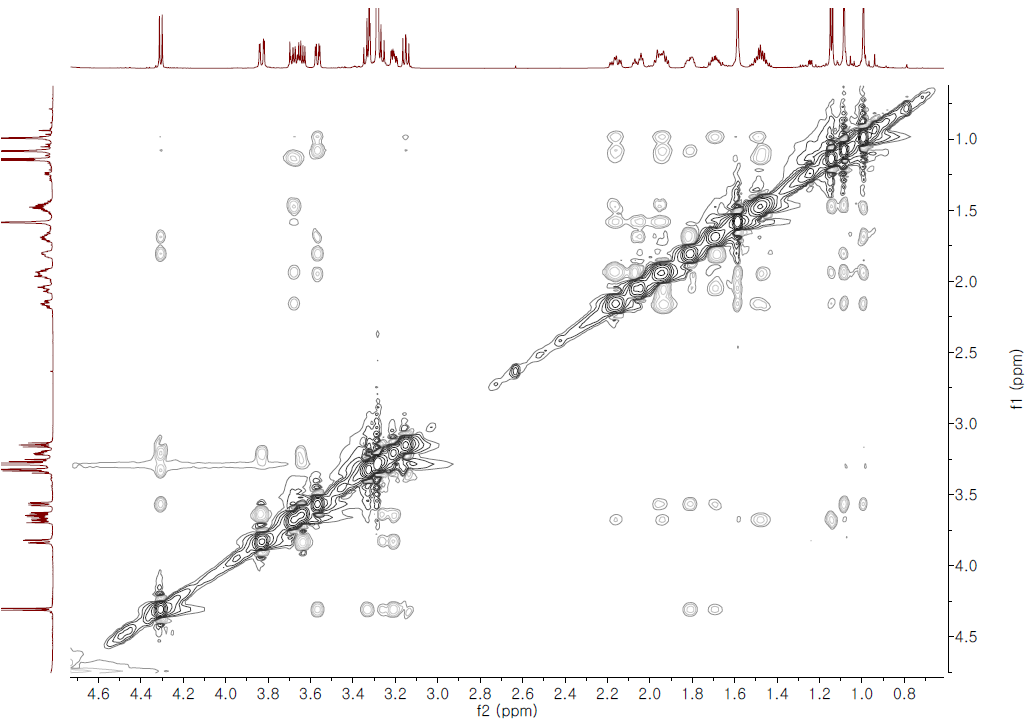


**Figure S12.** COSY spectrum (methanol-*d_4_*, 500 MHz) of compound **2.**


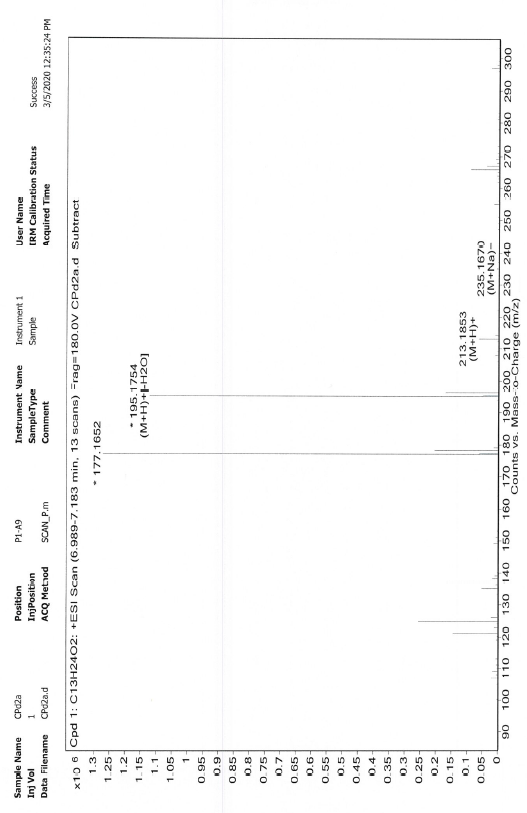

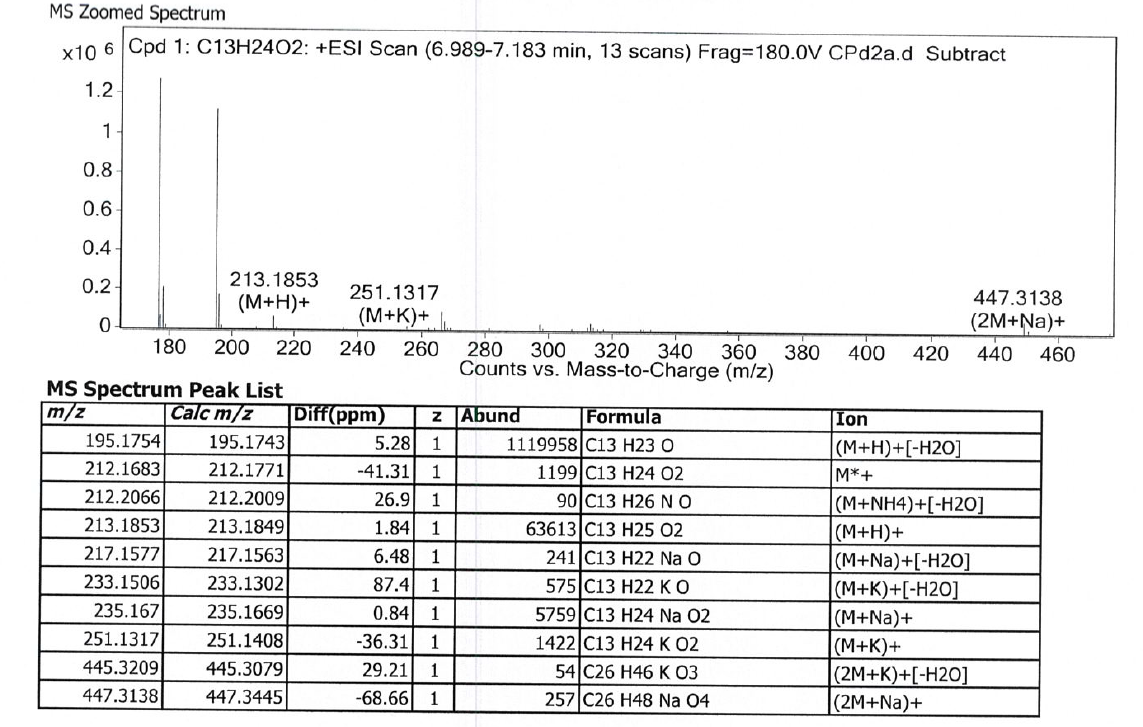


**Figure S13.** HRESIMS data of compound **2a.**


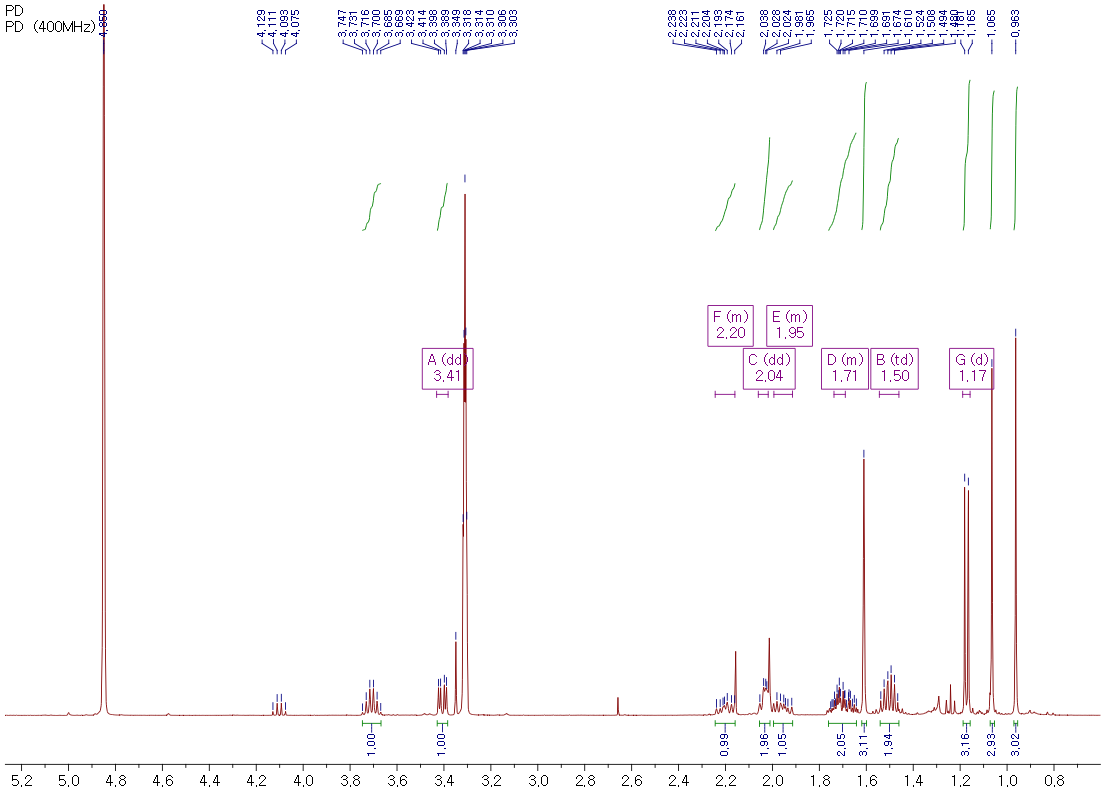


**Figure S14.** ^1^H NMR spectrum (methanol-*d_4_*, 400 MHz) of compound **2a.**


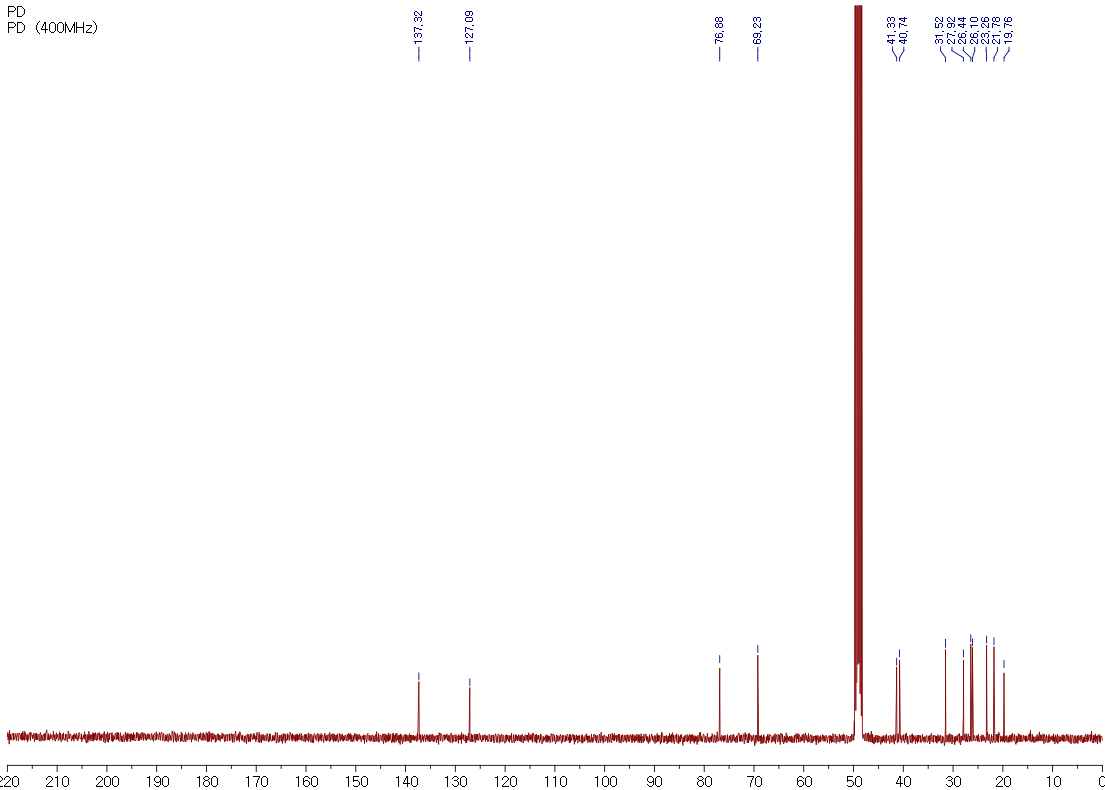


**Figure S15.** ^13^C NMR spectrum (methanol-*d_4_*, 100 MHz) of compound **2a.**


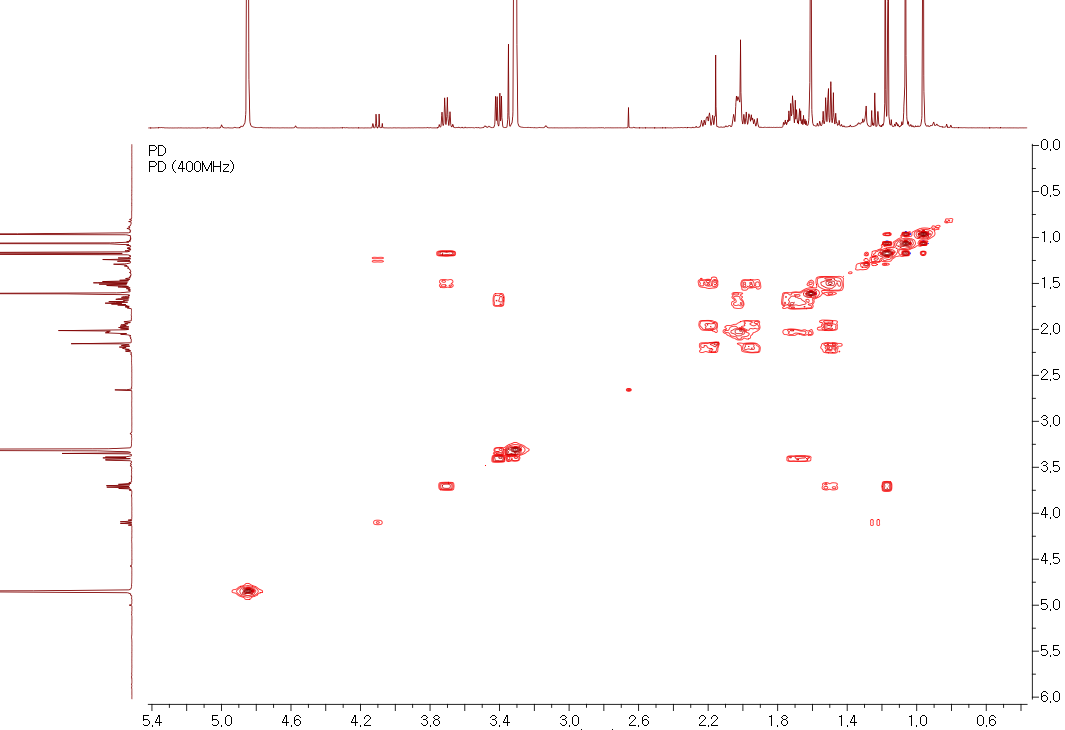


**Figure S16.** HSQC NMR spectrum (methanol-*d_4_*, 400 MHz) of compound **2a.**


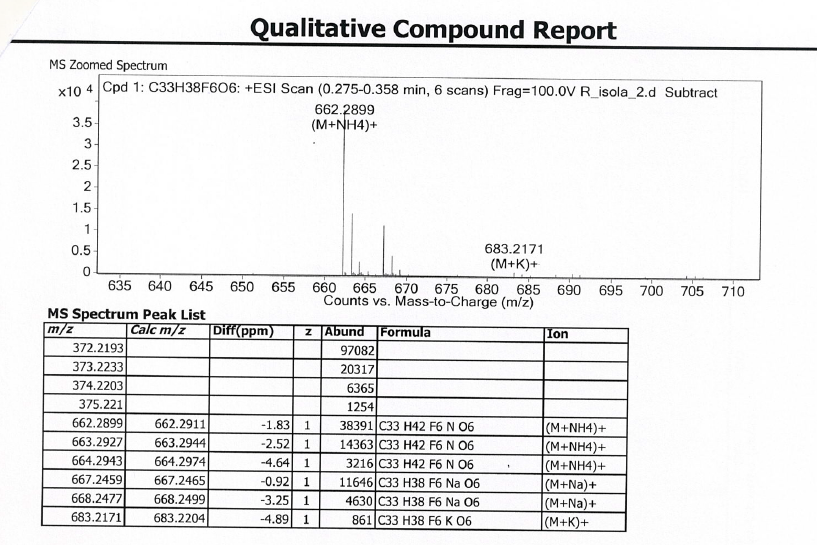


**Figure S17.** HRESIMS data of compound **2b.**


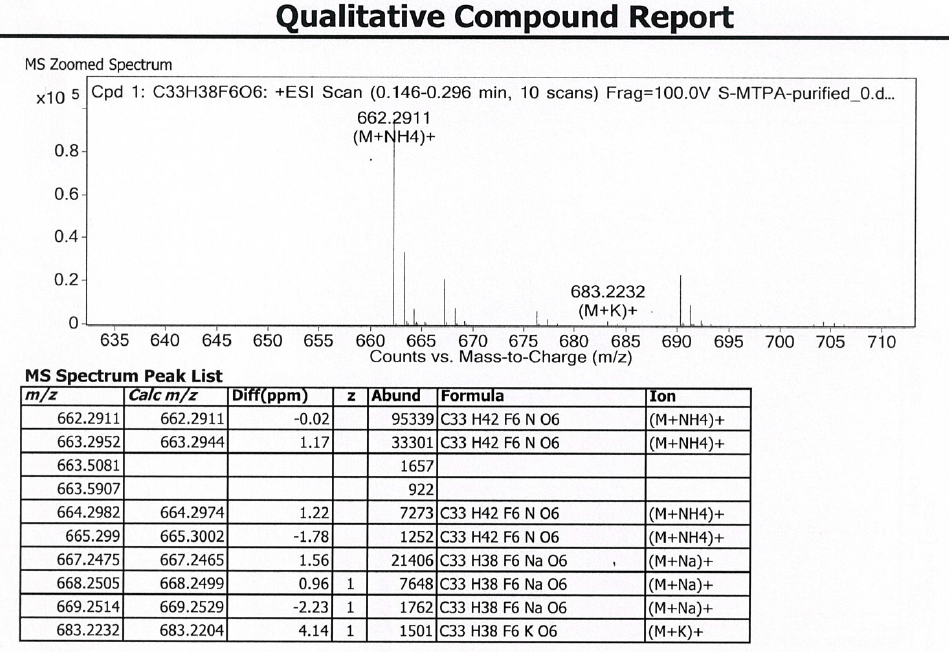


**Figure S18.** HRESIMS data of compound **2c.**


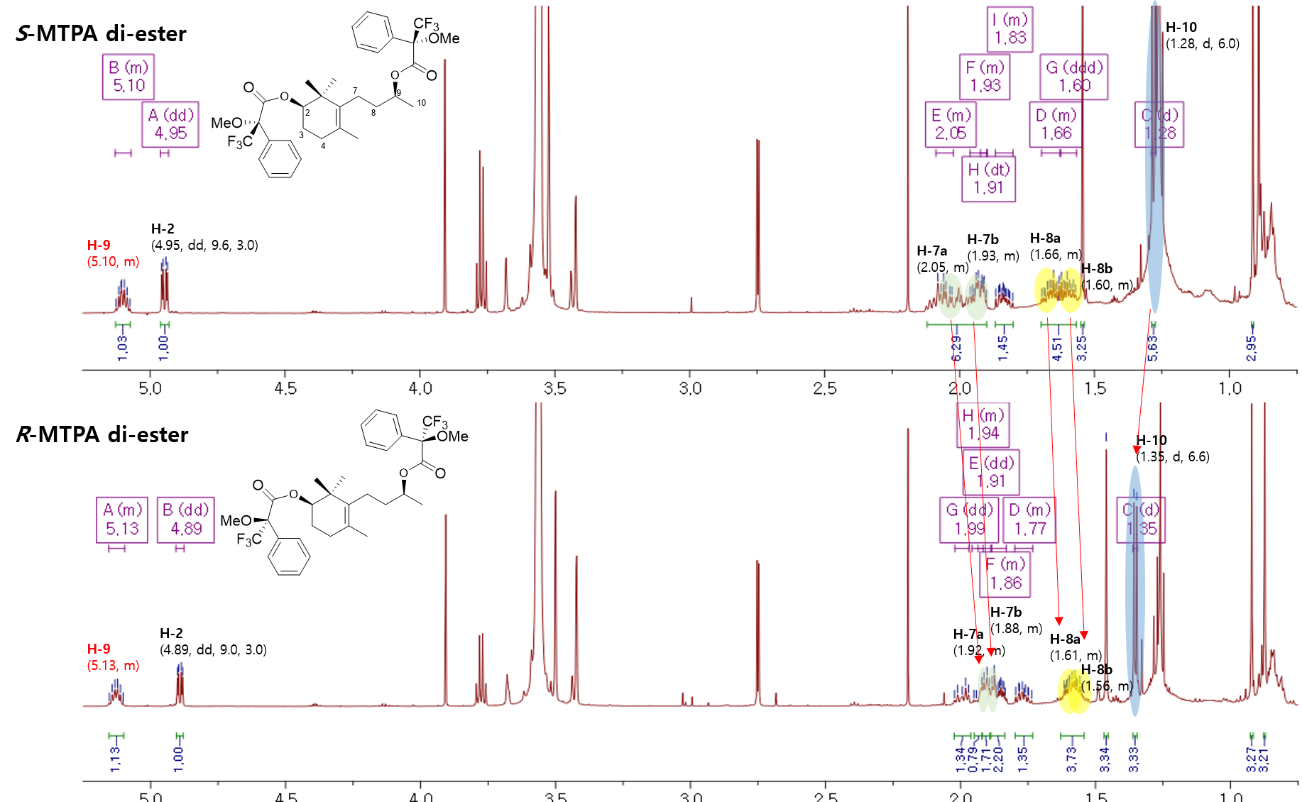


**Figure S19.** ^1^H NMR spectrum (CDCl_3_, 600 MHz) of compound **2b** and **2c.**


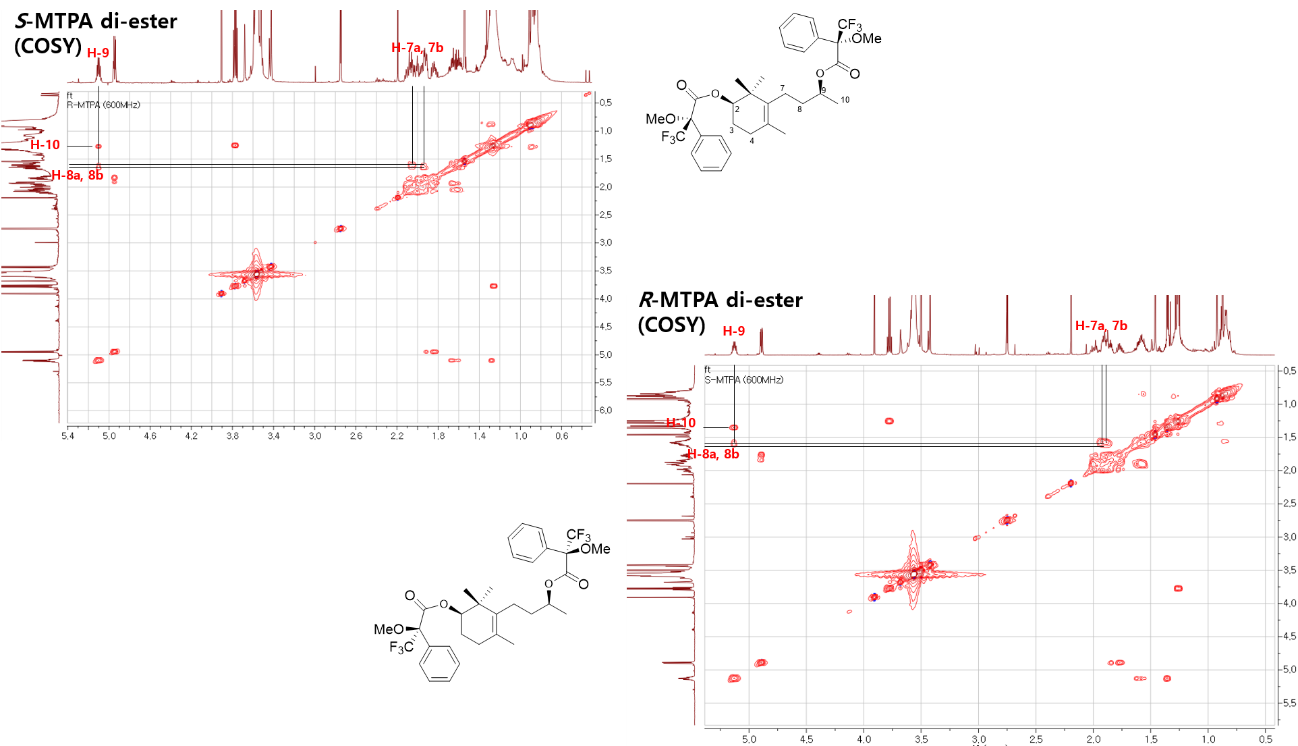


**Figure S20.** COSY spectrum (CDCl_3_, 600 MHz) of compound **2b** and **2c.**


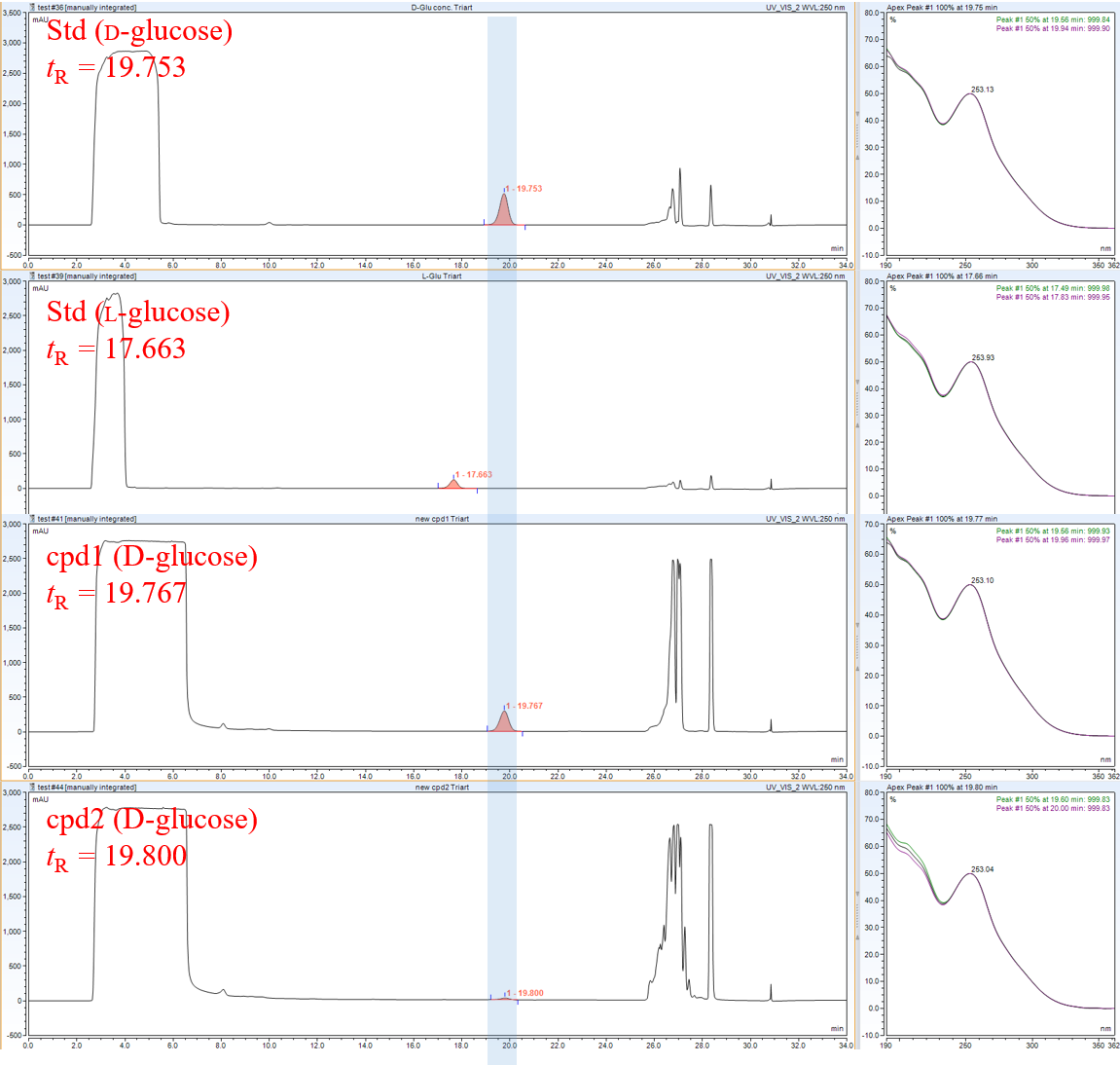


**Figure S21.** Comparison of chromatograms after sugar analysis for the determination of absolute configuration of sugar moiety in compound **1** and **2**.


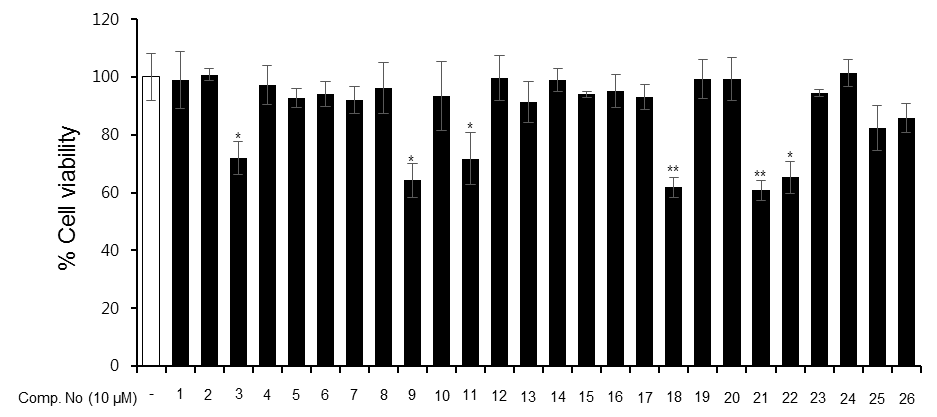


**Figure S22.** Percent cell viability of MDCK cells evaluated by the effects of all compounds (**1**−**26**) at 10 *µ*M using cytotoxicity assay.


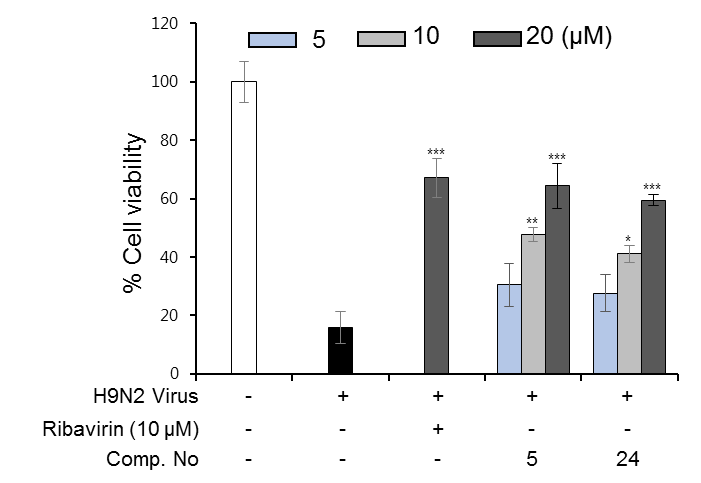


**Figure S23.** Inhibition of cytopathic effect of copmounds **5** and **24** against H9N2 virus.


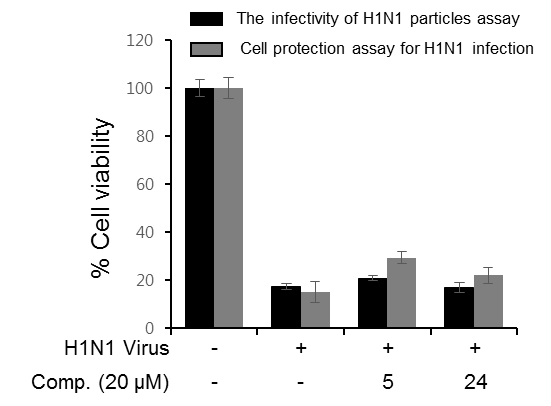


**Figure 24.** The effects of compounds **5** and **24** on the cell protection from viral infection and the H1N1 particles.


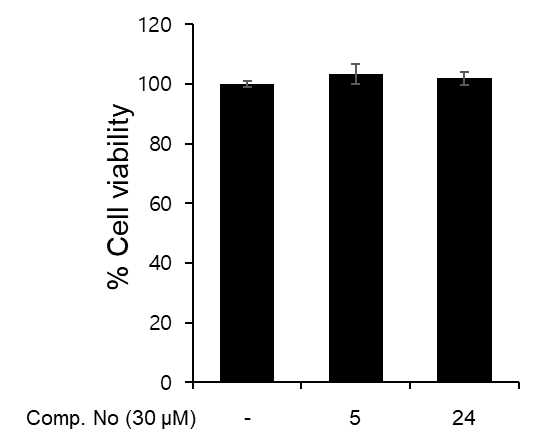


**Figure S25.** Percent cell viability of RAW 264.7 cells evaluated by the effects of compounds **5** and **24** at 30 *µ*M using cytotoxicity assay.


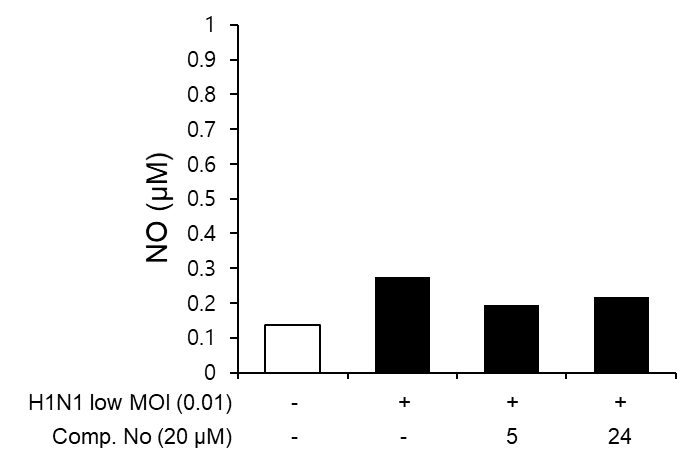


**Figure S26.** Effects of compounds **5** and **24** on the NO production in infected-RAW 264.7 cells with low MOI.


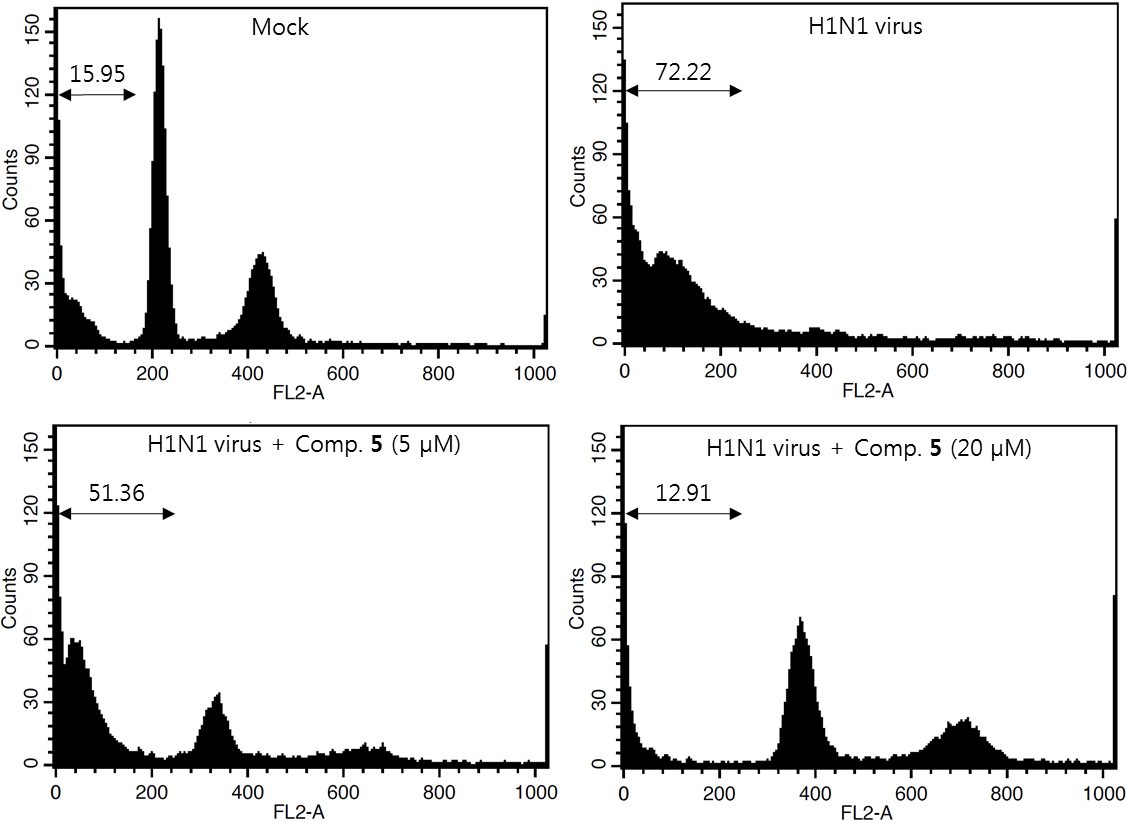


**Figure S27.** The effects of compound **5** on the cellular DNA contents 3 days after H1N1 virus.


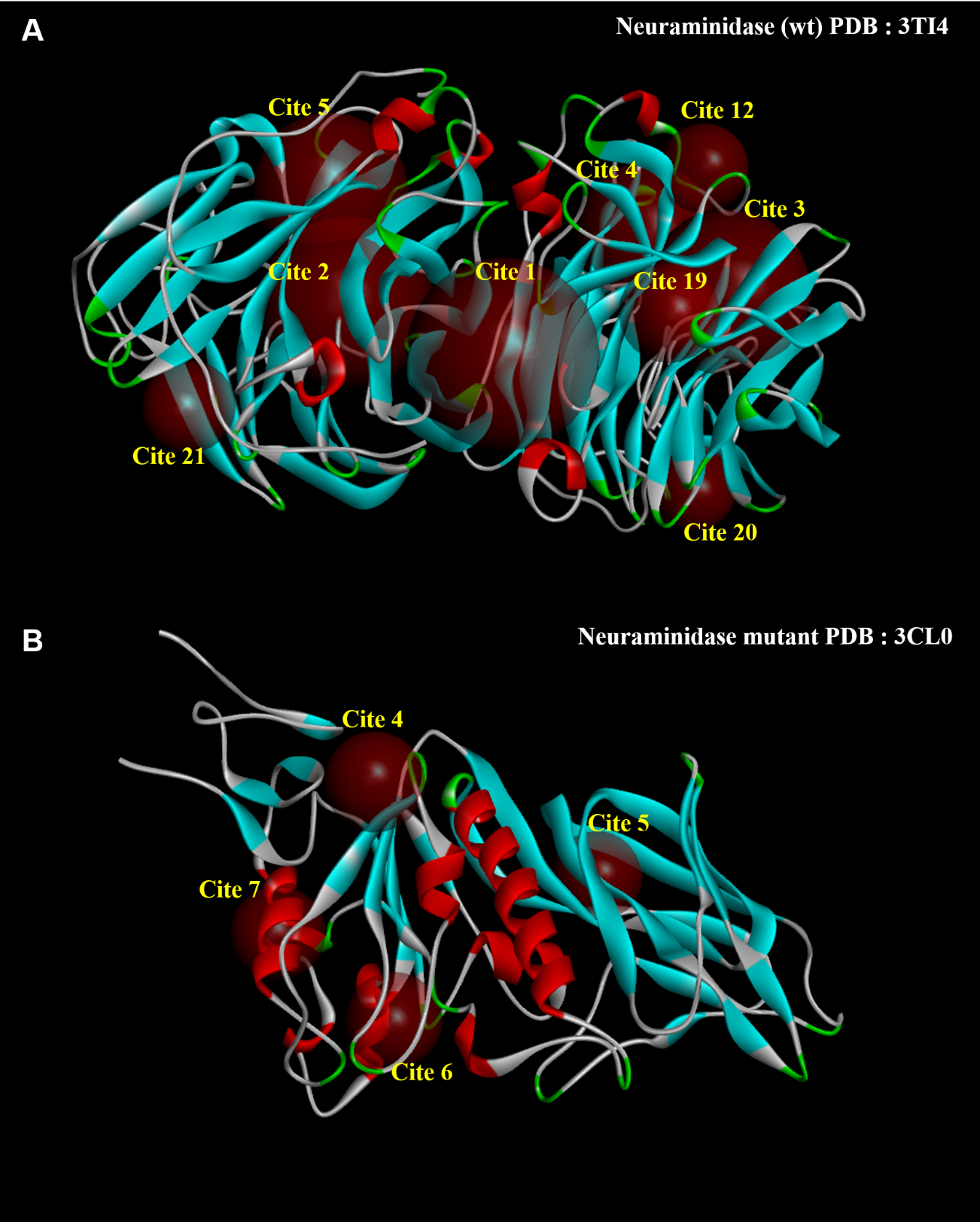


**Figure S28.** Images of global representation of NA surface with selected docking sites for compound **24**.

**Table S1.** CDOCKER and CDOCKER interaction energies of compounds **5** and **24** with nucleoprotein, PA-PB1 polymerase, neuraminidase, and neuraminidase mutant.

|  | **-CDOCKER energy**  **(kcal/mol)** | **-CDOCKER interaction energy (kcal/mol)** |
| --- | --- | --- |
|  | **Nucleoprotein** (PDB 3RO5) | |
| Binding site | Active site AC1 | |
| LHG | 15.5190 | 50.2549 |
| Comp. **5** | 17.2458 | 40.9050 |
|  | **PA-PB1 polymerase** (PDB 2ZNL) | |
| Binding site | Site 1 | |
| Comp. **5** | 7.5123 | 32.6849 |
|  | **Neuraminidase** (PDB 3TI4) | |
| Binding site | Site 1 | |
| Laninamivir octanoate | 37.1853 | 49.2786 |
| Comp. **24** | 38.5234 | 46.9320 |
|  | **Neuraminidase mutant** (PDB 3CL0) | |
| Binding site | Site 6 | |
| Oseltamivir | 27.3546 | 46.1881 |
| Comp. **24** | 24.4057 | 27.6933 |

**Table S2.** Molecular docking and interaction images of positive control with nucleoprotein, neuraminidase, and neuraminidase mutant.

|  | |
| --- | --- |
| **Nucleoprotein (PDB 3RO5)** | |
| Binding site | Active site AC1 |
| LHG | 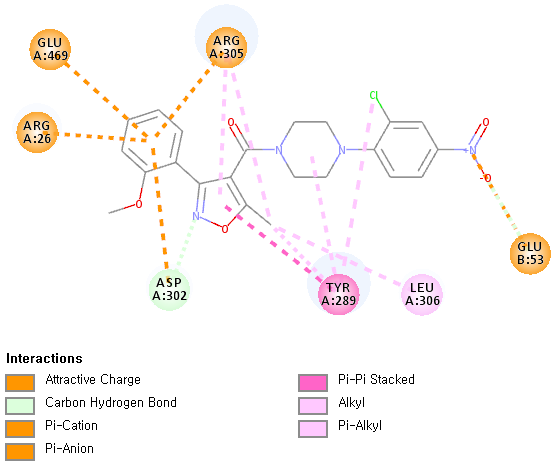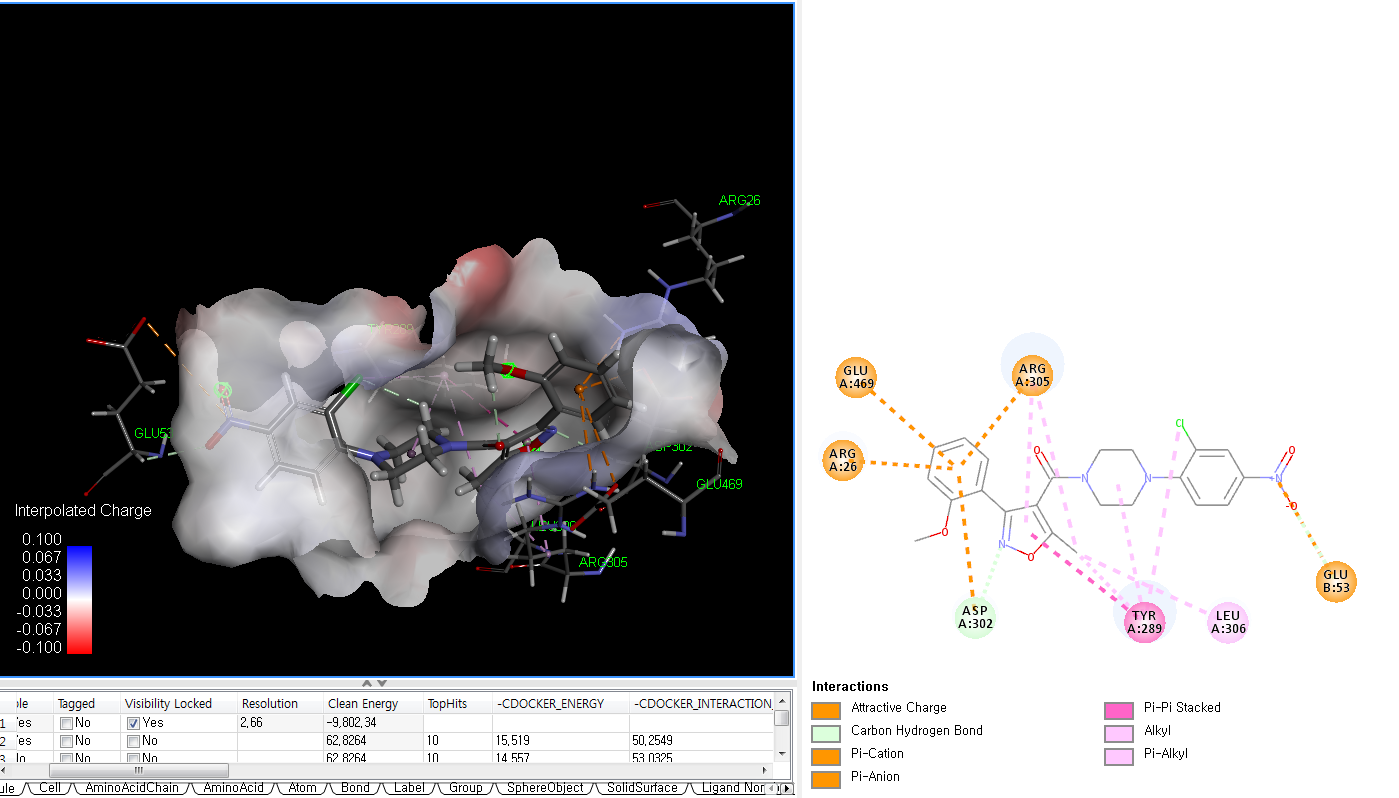 |
| **Neuraminidase (PDB 3TI4)** | |
| Binding site | Active site AC9 |
| Laninamivir octanoate | 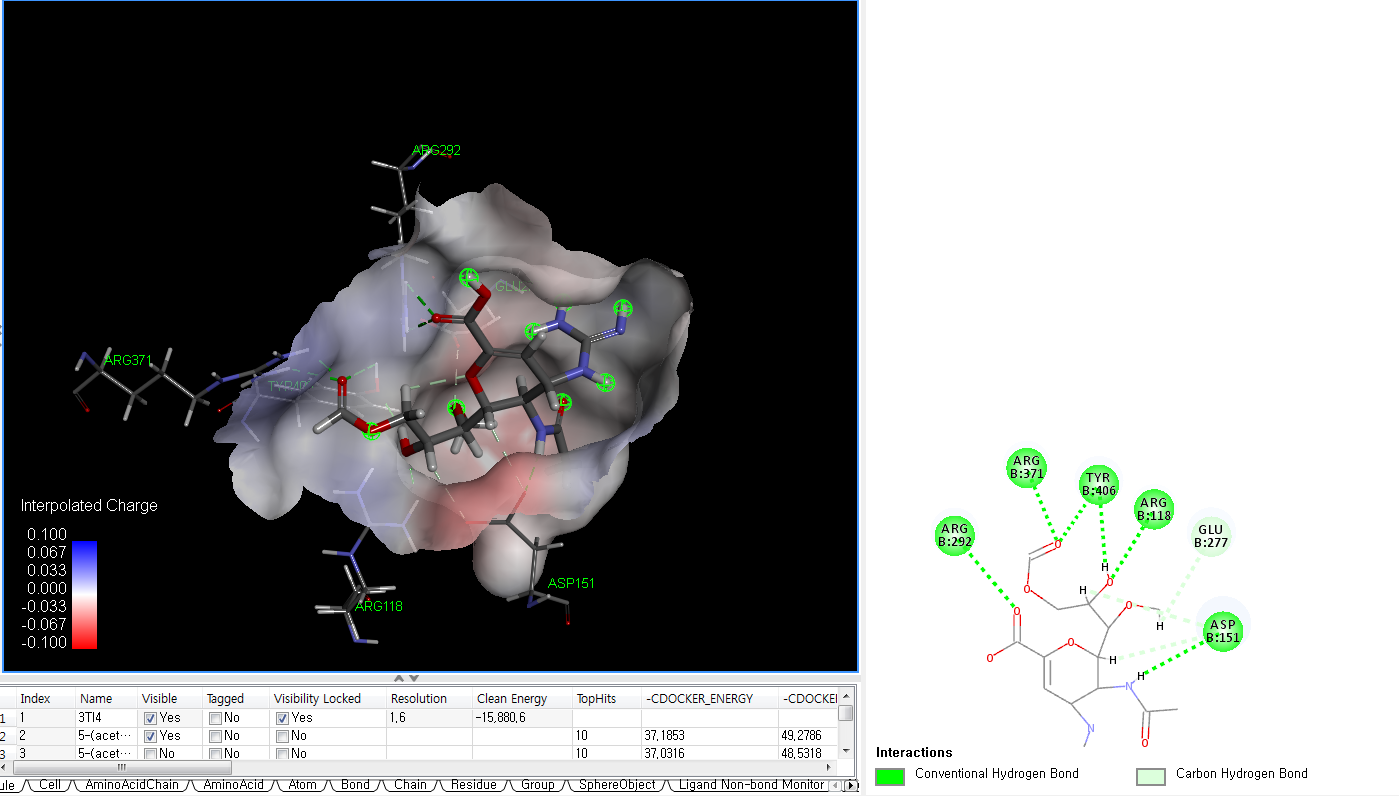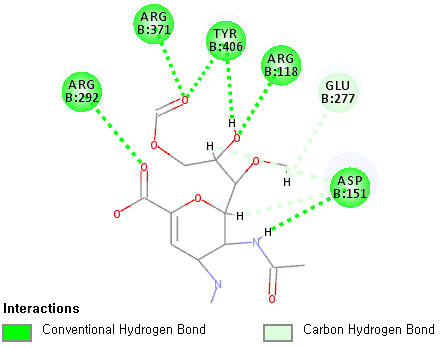 |
| **Neuraminidase mutant (PDB 3CL0)** | |
| Binding site | Active site AC2 |
| Oseltamivir | 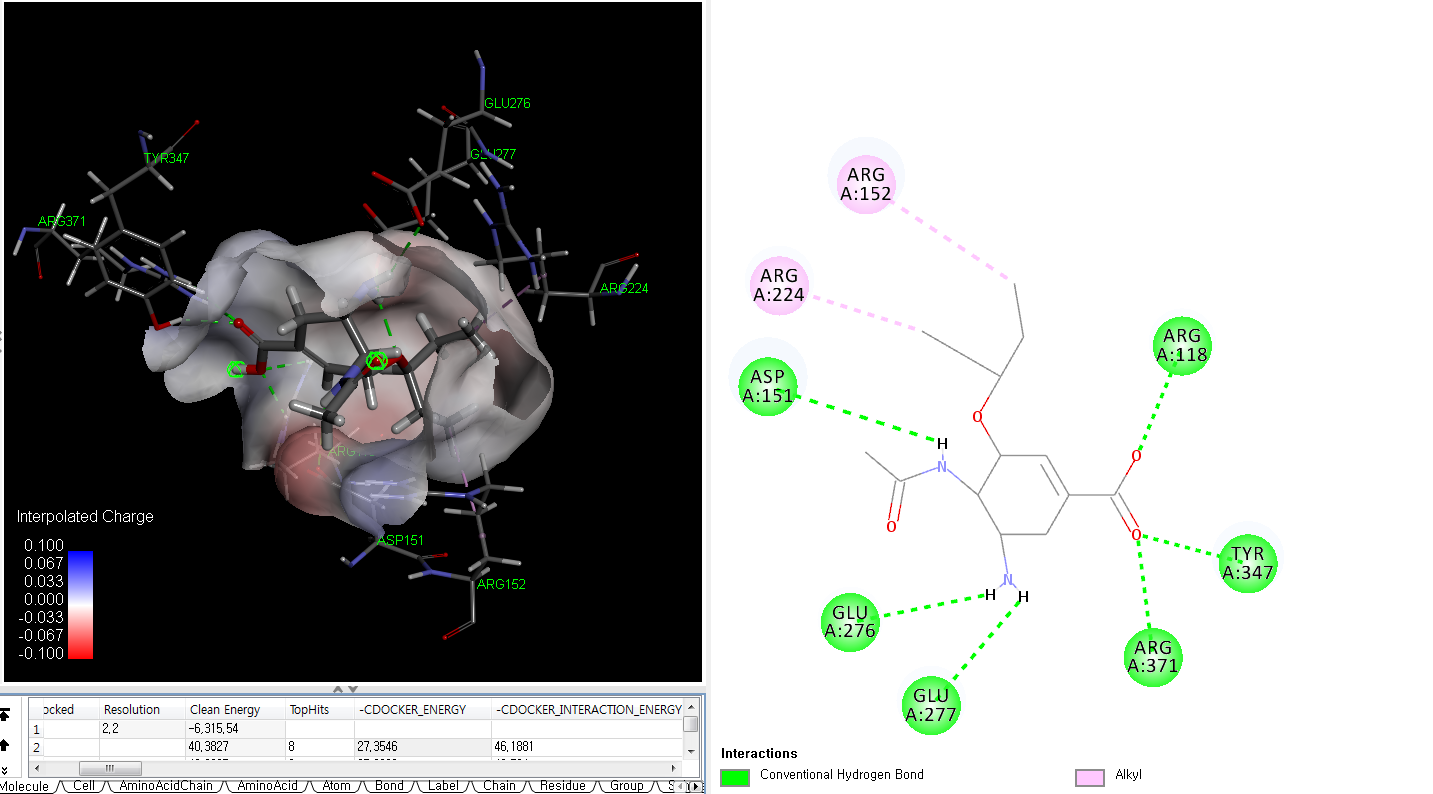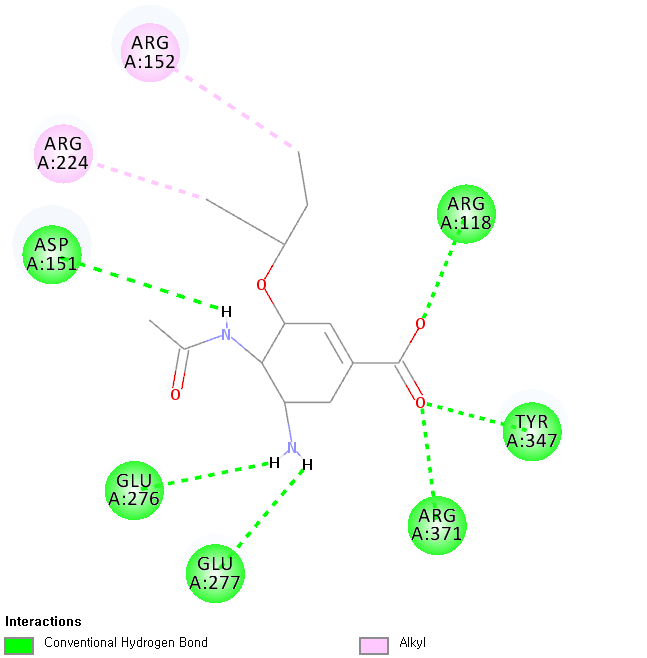 |

**Table S3.** CDOCKER and CDOCKER interaction energies of compound **24** with neuraminidase.

| **Binding site** | **-CDOCKER energy**  **(kcal/mol)** | **-CDOCKER interaction energy (kcal/mol)** |
| --- | --- | --- |
| 1 | 38.5234 | 46.9320 |
| 2 | 36.9229 | 45.5724 |
| 3 | 37.5789 | 46.4748 |
| 4 | 34.7293 | 40.2015 |
| 5 | 32.0745 | 40.3050 |
| 6 | -462.8790 | -135.8220 |
| 7 | -198.6140 | -65.4244 |
| 8 | -652.1040 | -154.7470 |
| 9 | -264.9350 | -79.2839 |
| 10 | -462.8790 | -135.8220 |
| 11 | Fail | Fail |
| 12 | 31.8543 | 35.6620 |
| 13 | Fail | Fail |
| 14 | Fail | Fail |
| 15 | Fail | Fail |
| 16 | Fail | Fail |
| 17 | Fail | Fail |
| 18 | Fail | Fail |
| 19 | 21.9884 | 29.7196 |
| 20 | 24.9146 | 32.1961 |
| 21 | 23.4295 | 27.3589 |

**Table S4.** CDOCKER and CDOCKER interaction energies of compound **24** with neuraminidase mutant.

| **Binding site** | **-CDOCKER energy**  **(kcal/mol)** | **-CDOCKER interaction energy (kcal/mol)** |
| --- | --- | --- |
| 1 | Fail | Fail |
| 2 | -68.8699 | -9.0374 |
| 3 | Fail | Fail |
| 4 | 21.7924 | 28.2420 |
| 5 | 24.4057 | 27.6933 |
| 7 | 16.7266 | 19.2418 |

**Table S5:** Primers used for real-time PCR

| **Primer** | **Sequence (5'−3')** |
| --- | --- |
| Neuraminidase - forward | CAG GCA TTC AAA TGG GAC TGT |
| Neuraminidase - reverse | GGC AGC TCA TTA AGG CCC TAT |
| Hemagglutinin - forward | GTT GAC ACA GTG CTC GAG AAG AA |
| Hemagglutinin - reverse | GGC TGT CTT CGA GCA GGT TAA |
| 18S - forward | AGC TAT CAA TCT GTC AAT CCT GTC |
| 18S - reverse | GCT TAA TTT GAC TCA ACA CGG GA |

**Supporting information NMR:** ^1^H NMR and ^13^C NMR of all isolated-compounds from the leaves and cotex of *Pinus densiflora*.

**Compound 1**: **(2*S*,9*S*)-2,9-dihydroxymegastigman-5-ene 9-*O*-*β*-ᴅ-glucopyranoside**

HRESI-MS: 419.2282 [M + HCOO]^-^ (calcd C_20_H_35_O_9_, 419.2287)

^1^H NMR (500 MHz, methanol-*d_4_*): 3.43 (dd, *J* = 3.0, 11.0 Hz, H-2), 2.04 (1H, m, H-3a), 1.79 (1H, m, H-3b), 2.05 (2H, m, H-4), 2.15 (1H, dt, *J* = 4.5, 13.0 Hz, H-7a), 2.00 (1H, m, H-7b), 1.51 (2H, m, H-8), 3.72 (1H, m, H-9), 1.18 (d, *J* = 6.0 Hz, H-10), 1.15 (3H, s, H-11), 1.05 (3H, s, H-12), 1.61 (3H, s, H-13); Glucose 4.35 (1H, d, *J* = 8.0 Hz, H-1), 3.22 (1H, t, *J*= 8.0 Hz, H-2), 3.36 (1H, m, H-3), 3.28 (1H, m, H-4), 3.26 (1H, m, H-5), 3.86 (1H, dd, *J* = 2.0, 11.5 Hz, H-6a), 3.68 (1H, dd, *J* = 5.5, 11.5 Hz, H-6b).

^13^C NMR (125 MHz, methanol-*d_4_*): 41.5 (C-1), 87.9 (C-2), 27.1 (C-3), 31.7 (C-4), 127.3 (C-5), 137.3 (C-6), 26.0 (C-7), 40.7 (C-8), 69.2 (C-9), 23.5 (C-10), 26.4 (C-11), 22.5 (C-12), 19.7 (C-13); Glucose 106.6 (C-1), 75.7 (C-2), 78.3 (C-3), 71.7 (C-4), 77.7 (C-5), 62.8 (C-6).

**Compound 2: (2*R*,9*S*)-2,9-dihydroxymegastigman-5-ene 9-*O*-β-D-glucopyranoside**

HRESI-MS: 419.2294 [M + HCOO]^-^ (calcd C_20_H_35_O_9_, 419.2287)

^1^H NMR (600 MHz, methanol-*d_4_*): 3.62 (dd, *J* = 3.0, 10.2 Hz, H-2), 1.86 (1H, m, H-3a), 1.74 (1H, m, H-3b), 2.11 (2H, td, *J* = 4.8, 16.8 Hz, H-4a), 2.02 (1H, m, H-4b), 2.21 (1H, dt, *J* = 4.8, 12.0 Hz, H-7a), 1.98 (1H, m, H-7b), 1.53 (2H, m, H-8), 3.73 (1H, m, H-9), 1.19 (d, *J* = 6.6 Hz, H-10), 1.14 (3H, s, H-11), 1.04 (3H, s, H-12), 1.64 (3H, s, H-13); Glucose 4.36 (1H, d, *J* = 7.8 Hz, H-1), 3.22 (1H, dd, *J* = 7.8, 9.0 Hz, H-2), 3.39 (1H, t, *J* = 9.0 Hz, H-3), 3.31 (1H, t, *J* = 9.0 Hz, H-4), 3.26 (1H, m, H-5), 3.88 (1H, dd, *J* = 1.8, 11.4 Hz, H-6a), 3.69 (1H, dd, *J* = 6.0, 11.4 Hz, H-6b).

^13^C NMR (150 MHz, methanol-*d_4_*): 40.6 (C-1), 83.0 (C-2), 23.9 (C-3), 31.3 (C-4), 127.1 (C-5), 137.5 (C-6), 26.1 (C-7), 40.8 (C-8), 69.2 (C-9), 23.3 (C-10), 26.7 (C-11), 22.7 (C-12), 19.7 (C-13); Glucose 101.8 (C-1), 75.1 (C-2), 78.3 (C-3), 71.9 (C-4), 77.8 (C-5), 63.0 (C-6).

**Compound 3: Dehydroabietic acid**

ESI-MS: 299.2 [M - H]^-^

^1^H NMR (300 MHz, CDCl_3_): 7.16 (1H, d, *J* = 7.2 Hz, H-11), 7.00 (1H, dd, *J* = 7.2, 1.8 Hz, H-12), 6.89 (1H, d, *J* = 1.8 Hz, H-14), 1.29 (3H, s, H- 18), 1.22 (6H, d, *J* = 6.6 Hz, H-16, 17), 1.22 (3H, s, H-20).

^13^C NMR (75 MHz, CDCl_3_): 38.1 (C-1), 18.7 (C-2), 36.9 (C-3), 47.6 (C-4), 44.7 (C-5), 21.9 (C-6), 30.2 (C-7), 134.8 (C-8), 146.9 (C-9), 37.0 (C-10), 124.2 (C-11), 124.0 (C-12), 145.8 (C-13), 127.0 (C-14), 33.6 (C-15), 24.2 (C-16), 24.2 (C-17), 185.0 (C-18), 16.4 (C-19), 25.3 (C-20).

**Compound 4: 12-hydroxydehydroabietic acid**

ESI-MS: 317.2 [M-H_2_O + H]^+^, 315.2 [M - H]^-^

^1^H NMR (300 MHz, CDCl_3_): 6.82 (1H, s, H-14), 6.62 (1H, s, H-11), 1.23 (6H, d, *J* = 6.7 Hz, H-16, 17), 1.27 (3H, s, H-18), 1.22 (3H, H-20).

^13^C NMR (75 MHz, CDCl_3_): 38.1 (C-1), 18.7 (C-2), 36.8 (C-3), 47.5 (C-4), 44.7 (C-5), 22.1 (C-6), 29.4 (C-7), 127.1 (C-8), 147.9 (C-9), 36.8 (C-10), 110.9 (C-11), 150.9 (C-12), 131.9 (C-13), 126.8 (C-14), 27.0 (C-15), 22.7 (C-16), 22.9 (C-17), 184.3 (C-18), 16.4 (C-19), 25.2 (C-20).

**Compound 5: 7*α*-methoxydehydroabietic acid**

ESI-MS : 331.2 [M + H]^+^

^1^H NMR (500 MHz, CDCl_3_): 7.17 (1H, d, *J* = 8.5 Hz, H-11), 7.18 (1H, d, *J* = 8.5 Hz, H-12), 7.14 (1H, s, H-14), 1.56 (6H, s, H-16, 17), 3.40 (3H, s, OMe), 2.86 (1H, m, H-15), 1.30 (3H, s, H-18), 1.22 (6H, d, *J* = 7.0 Hz, H-16, 17), 1.18 (3H, H-20).

**Compound 6: 15-hydroxydehydroabietic acid**

ESI-MS: 299.2 [M - H_2_O + H]^+^, 315.2 [M - H]^-^

^1^H NMR (300 MHz, CDCl_3_): 7.18 (3H, m, H-11, 12,14), 1.56 (6H, s, H-16, 17), 1.28 (3H, s, H-18), 1.22 (3H, H-20).

^13^C NMR (75 MHz, CDCl_3_): 38.0 (C-1), 18.7 (C-2), 36.8 (C-3), 47.5 (C-4), 44.7 (C-5), 21.9 (C-6), 30.3 (C-7), 134.8 (C-8), 146.0 (C-9), 37.0 (C-10), 124.2 (C-11), 122.1 (C-12), 147.9 (C-13), 125.0 (C-14), 72.6 (C-15), 31.7 (C-16), 31.7 (C-17), 184.7 (C-18), 16.4 (C-19), 25.2 (C-20).

**Compound 7: 16-nor-15-oxodehydroabietic acid**

ESI-MS: 301.2 [M + H]^+^, 299.2 [M - H]^-^

^1^H NMR (300 MHz, CDCl_3_): 7.68 (1H, d, *J* = 7.5 Hz, H-12), 7.62 (1H, s, H-14), 7.30 (1H, d, *J* = 7.5 Hz, H-11), 2.54 (3H, s, H-16), 1.56 (6H, s, H-16, 17), 1.28 (3H, s, H-18), 1.20 (3H, H-20).

^13^C NMR (75 MHz, CDCl_3_): 37.6 (C-1), 18.4 (C-2), 36.6 (C-3), 47.3 (C-4), 44.2 (C-5), 21.5 (C-6), 29.8 (C-7), 135.4 (C-8), 154.8 (C-9), 37.6 (C-10), 124.5 (C-11), 125.8 (C-12), 134.6 (C-13), 129.4 (C-14), 198.2 (C-15), 26.5 (C-16), 16.3 (C-18), 183.9 (C-19), 24.8 (C-20).

**Compound 8: Abieta-8,11,13,15-tetraen- 18-oic acid**

ESI-MS: 299.2 [M + H]^+^

^1^H NMR (300 MHz, CDCl_3_): 7.17 (3H, m, H-11, 12, 14), 5.30 (1H, brs, H-16a), 5.00 (1H, brs, H-16b), 2.10 (3H, brs, H-17), 1.28 (3H, s, H-18), 1.18 (3H, s, H-20).

**Compound 9: 7*α*-hydroxyabieta-8,11,13,15-tetraen-18-oic acid**

ESI-MS : 314.2 [M + H]^+^

^1^H NMR (500 MHz, CDCl_3_): 7.44 (1H, brs, H-14), 7.38 (1H, d, *J* = 8.0 Hz, H-12), 7.23 (1H, d, *J* = 8.0 Hz, H-11), 5.87 (1H, brs, H-16a), 5.06 (1H, brs, H-16b), 2.14 (3H, s, H-17), 1.28 (3H, s, H-18), 1.17 (3H, s, H-20).

**Compound 10: Karamatsuic acid**

ESI-MS: 317.2 [M + H]^+^

^1^H NMR (600 MHz, CDCl_3_): 6.93 (1H, dd, *J* = 7.8, 2.4 Hz, H-12), 6.91 (1H, d, *J* = 2.4 Hz, H-14), 6.77 (1H, d, *J* = 7.8 Hz, H-11), 1.22 (6H, d, *J* = 6.6 Hz, H- 16, 17), 1.11 (3H, s, H-18), 1.00 (3H, s, H-20).

^13^C NMR (150 MHz, CDCl_3_): 41.5 (C-1), 20.4 (C-2), 37.2 (C-3), 49.0 (C-4), 53.1 (C-5), 25.8 (C-6), 34.9 (C-7), 135.5 (C-8), 153.4 (C-9), 80.2 (C-10), 123.7 (C-11), 124.7 (C-12), 144.0 (C-13), 127.6 (C-14), 33.5 (C-15), 24.3 (C-16), 24.3 (C-17), 182.9 (C-18), 16.3 (C-19), 20.4 (C-20).

**Compound 11: Palustric acid**

ESI-MS: 303.2 [M + H]^+^, 301.2 [M - H]^-^

^1^H NMR (300 MHz, CDCl_3_): 5.39 (1H, brs, H-14), 1.20 (3H, s, H- 18), 1.02 (6H, d, *J* = 6.6 Hz, H-16, 17), 1.06 (3H, s, H-20).

^13^C NMR (75 MHz, CDCl_3_): 35.3 (C-1), 18.3 (C-2), 36.9 (C-3), 47.7 (C-4), 46.1 (C-5), 21.4 (C-6), 30.3 (C-7), 125.1 (C-8), 137.5 (C-9), 37.1 (C-10), 22.6 (C-11), 26.4 (C-12), 143.4 (C-13), 120.3 (C-14), 34.4 (C-15), 21.2 (C-16), 21.2 (C-17), 185.7 (C-18), 16.2 (C-19), 20.9 (C-20).

**Compound 12: 12-methoxy-7,13-abietadien-18-oic acid**

ESI-MS: 331.1 [M - H]^-^

^1^H NMR (300 MHz, CDCl_3_): 5.84 (1H, s, H-14), 5.48 (1H, brt, H-7), 3.77 (1H, t, *J* = 2.3 Hz, H-12), 1.21 (3H, s, H-18), 1.07 (3H, d, *J* = 6.9 Hz, H-16), 1.03 (3H, d, *J* = 6.9 Hz, H-18), 0.80 (3H, H-20).

^13^C NMR (75 MHz, CDCl_3_): 38.0 (C-1), 18.1 (C-2), 37.1 (C-3), 46.2 (C-4), 44.8 (C-5), 24.9 (C-6), 124.1 (C-7), 134.5 (C-8), 43.7 (C-9), 33.9 (C-10), 25.7 (C-11), 75.5 (C-12), 142.1 (C-13), 126.0 (C-14), 32.7 (C-15), 22.2 (C-16), 21.6 (C-17), 183.6 (C-18), 16.8 (C-19), 14.3 (C-20), 56.3 (OMe).

**Compound 13: 7-oxo-13*β*-hydroxyabiet-8(14)-en-18-oic acid**

ESI-MS: 317.2 [M - H_2_O + H]^+^ , 333.2 [M - H]^-^

^1^H NMR (300 MHz, CDCl_3_): 6.73 (1H, s, H-14), 1.23 (3H, s, H-18), 0.93 (3H, d, *J* = 6.9 Hz, H-16), 0.82 (3H, d, *J* = 6.9 Hz, H-18), 0.86 (3H, H-20).

^13^C NMR (75 MHz, CDCl_3_): 37.9 (C-1), 17.7 (C-2), 36.8 (C-3), 46.0 (C-4), 44.1 (C-5), 38.6 (C-6), 199.5 (C-7), 138.4 (C-8), 51.7 (C-9), 35.4 (C-10), 18.3 (C-11), 29.5 (C-12), 71.9 (C-13), 140.2 (C-14), 37.8 (C-15), 16.2 (C-16), 17.3 (C-17), 183.0 (C-18), 16.2 (C-19), 14.4 (C-20).

**Compound 14: 9,13*β*-epidioxy-8(14)-abieten-18-oic acid**

ESI-MS: 317.2 [M - H_2_O + H]^+^, 333.2 [M - H]^-^

^1^H NMR (300 MHz, CDCl_3_): 6.11 (1H, brs, H-14), 1.30 (3H, s, H- 18), 0.95 (3H, d, *J*= 6.6 Hz, H-16), 0.94 (3H, d, *J*= 6.6 Hz, H-17), 1.11 (3H, s, H-20).

^13^C NMR (75 MHz, CDCl_3_): 33.4 (C-1), 17.5 (C-2), 37.3 (C-3), 47.1 (C-4), 40.0 (C-5), 20.5 (C-6), 27.3 (C-7), 143.6 (C-8), 82.0 (C-9), 38.4 (C-10), 23.4 (C-11), 25.1 (C-12), 79.6 (C-13), 126.9 (C-14), 32.1 (C-15), 17.5 (C-16), 17.1 (C-17), 185.1 (C-18), 17.5 (C-19), 17.7 (C-20).

**Compound 15: 14*α*,15-epoxyabiet-9(11)-en-12-oxo-18-oic acid**

ESI-MS: 331.1 [M - H]^-^

^1^H NMR (300 MHz, CDCl_3_): 6.18 (1H, s, H-11), 5.24 (1H, d, *J* = 6.9 Hz, H-14), 2.99 (1H, d, *J* = 6.9 Hz, H-13), 1.60 (3H, s, H-16), 1.17 (3H, s, H-17), 1.14 (3H, s, H-18), 0.66 (3H, H-20).

^13^C NMR (75 MHz, CDCl_3_): 38.1 (C-1), 18.7 (C-2), 36.8 (C-3), 47.5 (C-4), 44.7 (C-5), 22.1 (C-6), 29.4 (C-7), 127.1 (C-8), 147.9 (C-9), 36.8 (C-10), 110.9 (C-11), 150.9 (C-12), 131.9 (C-13), 126.8 (C-14), 27.0 (C-15), 22.7 (C-16), 22.9 (C-17), 184.3 (C-18), 16.4 (C-19), 25.2 (C-20).

**Compound 16: 8(14)-podocarpen-13-on-18-oic acid**

ESI-MS: 277.2 [M + H]^+^ , 275.2 [M - H]^-^

^1^H NMR (300 MHz, CDCl_3_): 5.89 (1H, brs, H-14), 1.24 (3H, s, H-18), 0.85 (3H, H-20).

^13^C NMR (75 MHz, CDCl_3_): 38.4 (C-1), 18.1 (C-2), 36.8 (C-3), 47.2 (C-4), 48.0 (C-5), 24.2 (C-6), 35.3 (C-7), 165.0 (C-8), 51.8 (C-9), 38.4 (C-10), 20.5 (C-11), 36.8 (C-12), 200.0 (C-13), 126.4 (C-14), 184.1 (C-18), 17.0 (C-19), 15.7 (C-20).

**Compound 17: 8(14)-podocarpen-7,13-dion-18-oic acid**

ESI-MS: 291.2 [M + H]^+^ , 289.2 [M - H]^-^

^1^H NMR (300 MHz, CDCl_3_): 6.67 (1H, brs, H-14), 1.23 (3H, s, H-18), 0.80 (3H, H-20).

^13^C NMR (75 MHz, CDCl_3_): 37.8 (C-1), 17.6 (C-2), 36.7 (C-3), 45.9 (C-4), 43.8 (C-5), 38.6 (C-6), 198.9 (C-7), 151.6 (C-8), 51.7 (C-9), 35.7 (C-10), 22.8 (C-11), 37.9 (C-12), 199.9 (C-13), 130.4 (C-14), 180.1 (C-18), 16.3 (C-19), 14.9 (C-20).

**Compound 18: 4-*epi*-*trans*-communol**

ESI-MS: 289.2 [M + H]^+^

^1^H NMR (300 MHz, CDCl_3_): 6.33 (1H, dd, *J* = 10.8, 17.4 Hz, H-14), 5.41 (1H, t, *J* = 6.6 Hz, H-12), 5.03 (1H, d, *J* = 17.4 Hz, H-15a), 4.83 (1H, d, *J* = 17.4 Hz), 4.82 (1H, brs, H-17a), 4.46 (1H, brs, H-17b), 3.42 (1H, d, *J* = 10.8 Hz, H-19a), 3.11 (1H, d, *J* = 10.8 Hz, H-19b), 1.75 (3H, s, H-16), 0.77 (6H, s, H-18, 20).

^13^C NMR (75 MHz, CDCl_3_): 38.0 (C-1), 17.8 (C-2), 35.6 (C-3), 48.6 (C-4), 57.2 (C-5), 38.1 (C-6), 23.3 (C-7), 148.4 (C-8), 57.2 (C-9), 38.8 (C-10), 23.3 (C-11), 134.2 (C-12), 133.5 (C-13), 141.7 (C-14), 109.9 (C-15), 12.0 (C-16), 107.7 (C-17), 39.6 (C-18), 72.2 (C-19), 15.1 (C-20).

**Compound 19: 4-*epi*-*trans*-communal**

ESI-MS: 287.2 [M + H]^+^

^1^H NMR (300 MHz, CDCl_3_): 9.23 (1H, s, H-19), 6.32 (1H, dd, *J* = 10.8, 17.4 Hz, H-14), 5.40 (1H, t, *J* = 6.6 Hz, H-12), 5.03 (1H, d, *J* = 17.4 Hz, H-15a), 4.83 (1H, d, *J* = 17.4 Hz), 4.84 (1H, brs, H-17a), 4.49 (1H, brs, H-17b), 1.75 (3H, s, H-16), 1.05 (3H, s, H-18), 0.78 (3H, s, H-20).

^13^C NMR (75 MHz, CDCl_3_): 37.6 (C-1), 17.8 (C-2), 32.6 (C-3), 47.6 (C-4), 57.0 (C-5), 38.3 (C-6), 23.1 (C-7), 147.5 (C-8), 57.0 (C-9), 38.5 (C-10), 23.1 (C-11), 133.7 (C-12), 133.6 (C-13), 141.6 (C-14), 110.2 (C-15), 12.0 (C-16), 108.7 (C-17), 26.6 (C-18), 206.4 (C-19), 14.4 (C-20).

**Compound 20: 4-*epi*-*trans*-communic acid**

ESI-MS: 303.2 [M + H]^+^, 301.2 [M - H]^-^

^1^H NMR (300 MHz, CDCl_3_): 6.32 (1H, dd, *J* = 10.8, 17.4 Hz, H-14), 5.35 (1H, t, *J* = 6.6 Hz, H-12), 5.02 (1H, d, *J* = 17.4 Hz, H-15a), 4.85 (1H, d, *J* = 17.4 Hz), 4.84 (1H, brs, H-17a), 4.46 (1H, brs, H-17b), 1.73 (3H, s, H-16), 1.14 (3H, s, H-18), 0.77 (3H, s, H-20).

^13^C NMR (75 MHz, CDCl_3_): 38.1 (C-1), 18.4 (C-2), 37.6 (C-3), 47.5 (C-4), 57.0 (C-5), 26.6 (C-6), 37.1 (C-7), 147.8 (C-8), 49.4 (C-9), 38.8 (C-10), 23.0 (C-11), 133.6 (C-12), 133.5 (C-13), 141.6 (C-14), 109.9 (C-15), 16.4 (C-16), 108.1 (C-17), 14.7 (C-18), 184.8 (C-19), 11.8 (C-20).

**Compound 21: 3*β*-hydroxy-12,13(*E*)-biformen**

ESI-MS: 289.2 [M + H]^+^

^1^H NMR (300 MHz, CDCl_3_): 6.31 (1H, dd, *J* = 10.8, 17.4 Hz, H-14), 5.38 (1H, t, *J* = 6.6 Hz, H-12), 5.02 (1H, d, *J* = 17.4 Hz, H-15a), 4.85 (1H, d, *J* = 17.4 Hz), 4.81 (1H, brs, H-17a), 4.45 (1H, brs, H-17b), 3.24 (1H, dd, *J* = 4.2, 11.4 Hz, H-3), 1.72 (3H, s, H-16), 0.98 (3H, s, H-18), 0.77 (3H, s, H-19), 0.71 (3H, s, H-20).

^13^C NMR (75 MHz, CDCl_3_): 37.2 (C-1), 27.9 (C-2), 78.8 (C-3), 39.1 (C-4), 54.5 (C-5), 23.8 (C-6), 37.9 (C-7), 147.9 (C-8), 56.8 (C-9), 39.3 (C-10), 23.2 (C-11), 133.5 (C-12), 133.8 (C-13), 141.6 (C-14), 109.9 (C-15), 11.8 (C-16), 107.9 (C-17), 14.4 (C-18), 28.3 (C-19), 15.4 (C-20).

**Compound 22: 18*α*,3*α*-dihydroxy-12,13(*E*)-biformen**

ESI-MS: 287.2 [M - H_2_O + H]^+^, 349.2 [M + HCOO]^-^

^1^H NMR (300 MHz, CDCl_3_): 6.31 (1H, dd, *J* = 10.8, 17.4 Hz, H-14), 5.38 (1H, t, *J* = 6.6 Hz, H-12), 5.04 (1H, d, *J* = 17.4 Hz, H-15a), 4.87 (1H, d, *J* = 17.4 Hz), 4.82 (1H, brs, H-17a), 4.46 (1H, brs, H-17b), 3.7 (1H, d, *J* = 10.5 Hz, H-19a), 3.68 (1H, dd, *J* = 4.2, 11.1 Hz, H-3), 3.42 (1H, d, *J* = 10.5 Hz, H-19b), 1.74 (3H, s, H-16), 0.87 (3H, s, H-18), 0.77 (3H, s, H-20).

^13^C NMR (75 MHz, CDCl_3_): 37.0 (C-1), 27.6 (C-2), 76.5 (C-3), 42.4 (C-4), 49.0 (C-5), 24.0 (C-6), 37.8 (C-7), 147.7 (C-8), 56.9 (C-9), 39.3 (C-10), 23.4 (C-11), 133.7 (C-12), 133.7 (C-13), 141.7 (C-14), 110.1 (C-15), 12.0 (C-16), 108.2 (C-17), 11.5 (C-18), 71.7 (C-19), 15.0 (C-20).

**Compound 23: (13*S*)-15-hydroxylabd-8(17)-en-18-oic acid**

ESI-MS: 323.2 [M + H]^+^, 321.2 [M - H]^-^

^1^H NMR (300 MHz, CDCl_3_): 4.81 (1H, brs, H-17a), 4.51 (1H, brs, H-17b), 3.68 (2H, m, H-15) 1.15 (3H, s, H-18), 0.90 (3H, d, *J* = 6.3 Hz, H-16), 0.70 (3H, s, H-20).

^13^C NMR (75 MHz, CDCl_3_): 38.0 (C-1), 18.6 (C-2), 37.3 (C-3), 47.7 (C-4), 49.7 (C-5), 27.0 (C-6), 38.2 (C-7), 148.3 (C-8), 57.5 (C-9), 39.2 (C-10), 20.9 (C-11), 36.4 (C-12), 30.5 (C-13), 39.8 (C-14), 61.4 (C-15), 20.9 (C-16), 107.0 (C-17), 16.5 (C-18), 184.7 (C-19), 14.9 (C-20).

**Compound 24: Ampelopsin**

ESI-MS: 321.1 [M + H]^+^, 319.1 [M - H]^-^

^1^H NMR (300 MHz, methanol-*d_4_*): 6.51 (2H, s, H-2', 6'), 5.91 (1H, s, H-8), 5.88 (1H, s, H-6), 4.50 (1H, d, *J* = 11.4 Hz, H-3).

^13^C NMR (75 MHz, methanol-*d_4_*): 86.1 (C-2), 74.5 (C-3), 199.1 (C-4), 166.1 (C-5), 98.1 (C-6), 169.5 (C-7), 97.0 (C-8), 165.3 (C-9), 102.6 (C-10), 129.8 (C-1'), 108.8 (C-2'), 147.7 (C-3'), 135.7 (C-4'), 147.7 (C-5'), 108.8 (C-6').

**Compound 25: 5,7,4′-trihydroxy-3-methoxy-6-*C*-methylflavone**

ESI-MS: 315.1 [M + H]^+^, 313.1 [M - H]^-^

^1^H NMR (300 MHz, DMSO-*d­_6_*): 12.93 (1H, s, 5-OH), 7.92 (2H, d, *J* = 9.0 Hz, H-2', 6'), 6.93 (2H, d, *J* = 9.0 Hz, H-3', 5'), 6.50 (1H, s, H-8), 3.77 (3H, s, 3-OMe), 1.99 (3H, s, 6-Me).

^13^C NMR (75 MHz, DMSO-*d­_6_*): 155.3 (C-2), 137.5 (C-3), 177.8 (C-4), 158.2 (C-5), 106.5 (C-6), 162.2 (C-7), 92.7 (C-8), 154.0 (C-9), 103.8 (C-10), 120.6 (C-1'), 130.1 (C-2'), 115.6 (C-3'), 160.0 (C-4'), 115.6 (C-5'), 130.1 (C-6'), 59.7 (3-OMe), 7.4 (6-Me).

**Compound 26: 5,4′-dihydroxy-3.6,7-trimethoxy-8-*C*-methylflavone**

ESI-MS: 359.1 [M + H]^+^, 357.1 [M - H]^-^

^1^H NMR (300 MHz, DMSO-*d­_6_*): 12.60 (1H, s, 5-OH), 7.98 (2H, d, *J* = 8.7 Hz, H-2', 6'), 6.98 (2H, d, *J* = 8.7 Hz, H-3', 5'), 3.96 (3H, s, OMe), 3.88 (3H, s, OMe), 3.80 (3H, s, OMe), 2.06 (3H, s, 8-Me).

^13^C NMR (75 MHz, DMSO-*d­_6_*): 156.0 (C-2), 137.9 (C-3), 178.3 (C-4), 152.9 (C-5), 112.2 (C-6), 156.5 (C-7), 132.3 (C-8), 146.6 (C-9), 106.7 (C-10), 120.5 (C-1'), 130.1 (C-2'), 156.0 (C-3'), 160.4 (C-4'), 156.0 (C-5'), 130.1 (C-6'), 60.9 (3-OMe), 59.7 (6-OMe), 61.7 (7-OMe), 8.0 (8-Me).
